# Gene Expression Profiling in *Entamoeba histolytica* Identifies Key Components in Iron Uptake and Metabolism

**DOI:** 10.1371/journal.pone.0107102

**Published:** 2014-09-11

**Authors:** Nora Adriana Hernández-Cuevas, Christian Weber, Chung-Chau Hon, Nancy Guillen

**Affiliations:** 1 Institut Pasteur, Unité Biologie Cellulaire du Parasitisme, Paris, France; 2 INSERM U786, Paris, France; Universidade Federal do Rio de Janeiro, Brazil

## Abstract

*Entamoeba histolytica* is an ameboid parasite that causes colonic dysentery and liver abscesses in humans. The parasite encounters dramatic changes in iron concentration during its invasion of the host, with relatively low levels in the intestinal lumen and then relatively high levels in the blood and liver. The liver notably contains sources of iron; therefore, the parasite's ability to use these sources might be relevant to its survival in the liver and thus the pathogenesis of liver abscesses. The objective of the present study was to identify factors involved in iron uptake, use and storage in *E. histolytica.* We compared the respective transcriptomes of *E. histolytica* trophozoites grown in normal medium (containing around 169 µM iron), low-iron medium (around 123 µM iron), iron-deficient medium (around 91 µM iron), and iron-deficient medium replenished with hemoglobin. The differentially expressed genes included those coding for the ATP-binding cassette transporters and major facilitator transporters (which share homology with bacterial siderophores and heme transporters) and genes involved in heme biosynthesis and degradation. Iron deficiency was associated with increased transcription of genes encoding a subset of cell signaling molecules, some of which have previously been linked to adaptation to the intestinal environment and virulence. The present study is the first to have assessed the transcriptome of *E. histolytica* grown under various iron concentrations. Our results provide insights into the pathways involved in iron uptake and metabolism in this parasite.

## Introduction

*Entamoeba histolytica* is a unicellular eukaryote that causes amebiasis in humans after the ingestion of contaminated water or food-containing cysts. Trophozoites (the vegetative form of *E. histolytica*) released into the intestinal lumen by excystation may then penetrate the intestinal mucosal layer and cause colitis and bloody diarrhea [1]. In some cases, virulent trophozoites may also invade the liver and cause liver abscesses. Given that the host environments of the intestinal lumen and the liver are dramatically different, the parasite's abilities to adapt to these environments are considered to be crucial for its parasitic lifestyle [2]. *Entamoeba histolytica*'s ability to efficiently use iron sources in the liver might be relevant to the parasite's survival and the pathogenesis of liver abscesses. In fact, amebic proteins such as ferredoxin, alcohol dehydrogenase 2 [3] and superoxide dismutase [4] require iron in order to function. Moreover, the fact that trophozoites grown under low-iron concentration *in vitro* show low cell adherence and cytotoxic activities suggests that iron metabolism has a role in the pathogenesis of amebiasis [3], [5]. Despite iron's seemingly important roles in parasite survival and pathogenesis, the mechanisms underlying iron uptake, use and storage in *E. histolytica* have yet to be characterized.

Iron can enter eukaryotic cells by three major pathways: (i) uptake of ferrous iron (i.e., Fe^2+^); (ii) endocytosis of iron-binding proteins; and (iii) acquisition of heme. In the first pathway, ferric iron (i.e., Fe^3+^, the predominant form in the diet) is reduced to ferrous iron (Fe^2+^) by intestinal ferric reductase (for a review, see [6], [7]). Next, Fe^2+^ can be transported into enterocytes by the divalent metal transporter 1 (DMT1). In the second pathway, certain cell types acquire iron via transferrin receptor (TfR1)-mediated endocytosis of holotransferrin (transferrin (Tf) is the main iron carrier in plasma) [8]. Iron is transported into the cytoplasm by DMT1 after internalization of the transferrin-TfR1 complex. In the third pathway, iron is obtained from hemoglobin (Hb) or heme upon erythrocyte lysis. Heme and Hb are scavenged by hemopexin and haptoglobin, respectively. Hemoglobin's functions include oxygen transport from the lungs to the tissues, the removal of carbon dioxide and carbon monoxide from the body and the regulation of vascular tone through nitric oxide binding [7]. Heme is a prosthetic group in hemoproteins such as hemoglobin, myoglobin, cytochromes, catalases, and peroxidases. Enterocytes can internalize heme via the heme carrier protein 1 (HCP-1) transporter. Heme oxygenase-1 (HO-1) then cleaves the tetrapyrrole ring to yield α-biliverdin, carbon monoxide, and iron [6], [9]. Iron participates readily in redox reactions and produces reactive oxygen species (ROS) [10]. Since free iron is toxic to the cell, it is rapidly bound to ferritin and/or released into the circulation via the ferrous iron transporter ferroportin [11]. In the mitochondria, iron is used to synthesize heme and iron-sulfur (Fe-S) clusters. In microbial pathogens, heme acquisition and the endocytosis of iron-binding proteins are the main routes for iron uptake in host environments. Mechanisms of iron uptake have been best studied in bacteria and include (i) receptor-mediated binding of transferrin, lactoferrin and Hb, (ii) secretion of Fe^3+^-chelating siderophores and (iii) hemophores (heme-chelating proteins that remove heme from diverse sources). The iron-siderophore and heme-hemophore complexes enter the cell via specialized receptors (for a review, see [12]).

It has been reported that *E. histolytica* is able to obtain iron from the bacterial flora and the host's iron-binding proteins (e.g., transferrin, hemoglobin, ferritin, and lactoferrin). These proteins are usually released when the amoeba lyses and phagocytes cells [13]. However, the fact that hemolytic activities have been detected in *E. histolytica* trophozoites [13] suggests that the parasites might be able to lyse red blood cells (RBCs) and liberate the host's heme via an non-phagocytic route. Furthermore, it has been suggested that two heme-binding proteins secreted by *E. histolytica* facilitate the scavenging of host heme [14]. Little is known about iron uptake, utilization and storage pathways in *E. histolytica*, with the exception of a few enzymes (i.e., NifS, NifU and rubrerythrin) involved in Fe-S cluster pathways [15], [16].

The objective of the present study was to identify candidate factors involved in iron uptake, use and storage mechanisms in *E. histolytica.* We compared the transcriptome of *E. histolytica* trophozoites grown *in vitro* under four conditions: (i) normal iron concentrations (around 169 µM iron); (ii) low iron concentrations (around 123 µM); (iii) iron deficiency (91 µM) and (iv) iron deficiency with Hb replenishment. Considering the parasite's hemolytic activities and its phagocytosis of RBCs, Hb replenishment was used to mimic the transition from low iron levels in the intestine to high (Hb-derived) iron levels in the blood and liver. The differentially expressed candidate genes coded for proteins with a function in iron uptake (i.e., major facilitator transporters (MFTs) and ATP-binding cassette (ABC) transporters, which share homology with bacterial siderophores or heme transporters) or were homologous to important genes involved in heme biosynthesis and degradation in bacteria, plants, and humans (e.g., S-adenosylmethionine synthetase, glutamyl-tRNA synthetase, and monooxygenases). This study is the first to have assessed the transcriptome of *E. histolytica* grown with various iron concentrations and enabled us to identify candidates involved in iron uptake and metabolism in the parasite.

## Methods

### *Entamoeba histolytica* culture

The axenic, virulent *E. histolytica* strain HM1:IMSS was cultured in the TYI-S-33 medium at 37°C [17]. The TYI-S-33 medium contains 15% serum, 78 µM ammonium ferric citrate (AFC) and vitamins (Table S1 in File S1). The serum and peptone contribute 90.7 µM iron, meaning that the “normal iron” condition corresponds to ≈169 µM iron. In the “low iron” condition, 2×10^3^ amoebae mL^−1^ grown in normal iron medium were grown for 6 days in modified TYI-S-33 medium (Table S1 in File S1) supplemented with just 32 µM AFC (yielding a final iron concentration of around 123 µM). The medium was changed at 72 and 96 hrs post-inoculation. The trophozoites were grown for 3 days in low iron and then were supplemented with 52 µM Hb (Sigma, H7379, France) for three days to determine the expression level of modulated genes by qRT-PCR assay. To obtain trophozoites adapted to “iron deficiency”, trophozoites were grown for one month in TYI-S-33 medium in which the AFC supplementation was reduced in a step-wise manner every three days (from 80 µM AFC to 40 µM, 30 µM, 20 µM, 10 µM and 0 µM AFC). Hence, the “iron deficiency” condition corresponds to TYI-S-33ΔFe medium that contained only the iron contributed by serum and peptone (90.7 µM) (Table S1 in File S1). Trophozoites were growth for an additional seven weeks in iron-deficient medium prior to the extraction of RNA of transcriptomic analysis. Lastly, the iron-deficiency–adapted strain grown in TYI-S-33ΔFe was then supplemented with 84 µM Hb for 2 hours at 37°C (i.e. the “iron deficiency + Hb” condition). The number of amoebae was counted every day for five days using a hemocytometer (Neubauer, Germany). Reported values correspond to the mean of experiments performed in triplicate.

### Quantification of iron in the culture medium

The ferrozine method [18] was used to quantify iron in the TYI-S-33 and TYI-S-33ΔFe media. Briefly, 100 µl of TYI-S-33 or TYI-S-33ΔFe medium were mixed with 100 µl of 10 mM HCl (the solvent of the iron standard FeCl_3_) and 100 µl of the iron-releasing reagent (a freshly prepared solution containing equal volumes of 1.4 M HCl and 4.5% (w/v) KMnO_4_) and were incubated for 2 hrs at 60°C. Next, 30 µl of the iron-detection reagent (6.5 mM ferrozine, 6.5 mM neocuproine, 2.5 M ammonium acetate, and 1 M ascorbic acid) were added to the samples. After a 30 min incubation, 280 µl aliquots of the sample solutions were transferred into the wells of a 96-well plate. Absorbance at 550 nm was measured in a microplate reader. Each sample's iron content was calculated with respect to a standard curve established with 0 to 300 µM FeCl_3_ in the same reaction mixture. Reported values correspond to the mean of experiments performed in triplicate.

### RNA preparation and microarray experiments

RNA prepared from trophozoites grown in the reference condition (normal iron) and the query conditions (i.e., iron deficiency, iron deficiency + Hb and low iron) was used for microarray analyses. RNA was obtained from three independent cultures grown with at least a one-week interval (i.e. three biological replicates). The differentially expressed genes mentioned below refer to genes expressed at significantly different levels in one or more of the three query conditions, relative to the reference condition. RNA was purified from 4×10^6^ trophozoites using Trizol reagent (Invitrogen by LifeTechnologies Corp., USA), according to the manufacturer's protocol. The quality and integrity of the purified RNA was checked with spectrophotometry, electrophoresis on 0.8% agarose gels, and capillary electrophoresis in a Bioanalyser 2100 (Agilent Technologies, Les Ulis, France). The RNA was reverse-transcribed using Superscript III Reverse Transcriptase (45-0039, Invitrogen by LifeTechnologies Corp.) [19], according to the manufacturer's protocol. Cy3- or Cy5-labeled cDNA obtained from amoeba grown under each of the query conditions was cohybridized with Cy3- or Cy5-labeled cDNA from amoebae grown in the reference condition on EH2008 oligomicroarrays [19], [20]. Standard dye-swap hybridizations were carried out. The EH2008 microarray is a custom-designed microarray developed by our group. It contains oligonucleotides covering the open reading frames from *E. histolytica* genome [21]. After pooling data from technical and biological replicates, differential expression analysis was carried out using both a paired Student's *t*-test and the variance estimating method in the VarMixt software package [22]. The raw *P*-values were adjusted using the Benjamini and Yekutieli method [23], which monitors the false discovery rate. Differentially expressed genes were considered to be those with a Benjamini and Yekutieli *P*-value <0.05 and a ≥2-fold change in expression level. Complete experimental details and data sets are available in the “ArrayExpress” MIAME-based database (www.ebi.ac.uk/arrayexpress/) with the accession number E-MTAB-1158.

### Quantitative real-time PCR

RNA was reverse-transcribed with Superscript III Reverse Transcriptase (45-0039, Invitrogen by LifeTechnologies Corp., USA), according to the manufacturer's protocol and using the qRT-PCR primers listed in Table S7 in File S1. qRT-PCRs were performed on the RNA samples used in the microarray assay. After reverse transcription, qRT-PCRs were carried out using an ABI Prism 7900HT system (Applied BioSystems by LifeTechnologies Corp.). Reactions were performed in a 15 µl volume containing 667 nM of each primer, 1× PCR SYBR Green Master Mix (4309155, Applied Biosystems by LifeTechnologies Corp., USA) and 1 µg of template cDNA. A control curve (with 10-fold serial dilutions of cDNA) was used to check the amplification efficiency. The α-tubulin transcript was used to normalize cycle threshold (Ct) values because its expression levels were stable throughout all our microarray experiments. For each biological triplicate, the mean relative concentration was divided by the mean of the values obtained for the normal iron condition. This ratio represents the change in the tested gene's mRNA abundance under the different iron concentrations, relative to the normal iron condition.

### Identification of iron-responsive-element-like structures in *Entamoeba histolytica*

The Searching for Iron-Responsive Elements (SIREs) web server (http://ccbg.imppc.org/sires/index.html) was used to identify iron-responsive-element (IRE)-like sequences in the set of *E. histolytica* mRNAs. The server's predictions have high, medium, and low levels of stringency. High- and medium-stringency predictions are mainly based on IREs that have been well characterized *in vivo* and/or *in vitro*, whereas low-level stringency predictions are based on mRNAs that interacted with novel iron-regulatory proteins (IRPs) in a recent genome-wide study. Some of these predictions have been validated *in vitro* but not *in vivo* [24]. The SIREs program also considers other criteria, including the motif type, apical loop, the nucleotide at position 25, the number of UG pairs, and the free energy of the stem-loop structures. The putative 5′- and 3′-UTRs (defined as the 100 nucleotides on each side of the coding sequences) and coding sequences (n = 8306, based on AmoebaDB version 1.3) were submitted for analysis using default parameters. The mouse ferredoxin sequence (NM_010239, which contains an IRE sequence) was included as a positive control in each submitted group.

## Results

### The growth conditions and their effects on trophozoites

The ferrozine method was used to quantify the iron levels in the TYI-S-33 and TYI-S-33ΔFe media (Table S1 in File S1). We measured the iron concentration in TYI-S-33 medium to be 168.9 µM±5.5 with 78 µM AFC supplementation and 90.7 µM±6.1 in the absence of supplementation (Table S1 in File S1). Serum and peptone contribute ≈55 µM and ≈40 µM, respectively (Table S1 in File S1). The iron concentration in low-iron medium is approximately 123 µM (Table S1 in File S1). Under low-iron conditions, the initial mortality rate was ≈30%. However, four days later, the surviving trophozoites grew at much the same rate as in normal TYI-S-33 medium. The iron concentration in the TYI-S-33ΔFe medium (90.7 µM) was supplemented with 84 µM Hb (Table S1 in File S1). In the iron deficiency condition, trophozoites showed a longer lag growth phase but achieved a normal growth rate after one day (Figure S1 in File S1).

To identify changes in gene expression in trophozoites grown with different iron concentrations, total RNA was purified and analyzed with microarrays. We analyzed the gene expression profiles of trophozoites grown with different iron concentrations in order to mimic the scenarios probably faced by *E. histolytica* during infection: adaptation to the low iron concentration in the intestine (mimicked by TYI-S-33 medium supplemented with only 32 µM AFC). We selected 32 µM AFC (Table S1 in File S1) because this was the threshold concentration for a subnormal growth rate (since trophozoites grew as well in TYI-S-33 medium supplemented with 40 µM AFC as they do in TYI-S-33 medium supplemented with around 78 µM AFC). Trophozoites initially grow in normal TYI-S-33 medium were able to adapt to and grow in iron-deficient medium (via stepwise reductions in the magnitude of AFC supplementation, see the Methods). These adapted trophozoites were then supplemented with Hb as an iron source for 2 hours, in order to mimic the higher iron concentrations encountered in the blood and liver blood. *E. histolytica* is known to use Hb as an iron source *in vitro* [13].

### Genes differentially expressed under different iron conditions

The transcriptomes of trophozoites exposed to different iron concentrations were evaluated using our previously designed genome-wide microarray [21]. Differentially expressed genes refer to genes expressed at significantly different levels under low-iron, iron deficiency and/or iron deficiency + Hb conditions when compared with normal iron conditions. The gene expression profile of trophozoites in the iron deficiency condition revealed transcripts modulated upon adaptation to iron deficiency, whereas the gene expression of trophozoites in the iron deficiency + Hb condition revealed transcripts modulated upon uptake and further degradation of Hb (which is expected to rapidly increase intracellular iron levels). A comparison of the gene expression profiles under low-iron, iron deficiency and iron deficiency + Hb conditions with the profile in the normal iron condition revealed a total of 224 transcripts with significantly modulated (*P*<0.05 and at least a two-fold change) expression levels. The distribution and overlap of these transcripts under the various conditions is represented as a Venn diagram (Figure S2 in File S1). In iron deficiency, the transcriptome did not differ greatly from that seen with normal iron levels. Only a few genes were modulated (9 upregulated transcripts and 11 downregulated transcripts; Table S2 and Figure S2 in File S1), suggesting that the trophozoites were able to adapt to iron deficiency. Addition of Hb revealed 107 upregulated transcripts and 50 downregulated transcripts (Table S3 and Figure S2 in File S1). In cells grown under low-iron conditions, 34 transcripts were upregulated and 46 were downregulated (Table S4 and Figure S2 in File S1). Of the 42 genes extracted from AmoebaDB using the keyword “iron”, 2 (5%) were downregulated in iron deficiency and 10 (24%) were upregulated however after Hb supplementation.

The microarray results were validated by quantitative real-time PCR assays (Table 1). We selected 6 transcripts that were downregulated under low-iron conditions and 5 that were upregulated. The qRT-PCR data agreed with the microarray results in all cases. Putative functions for the proteins encoded by the identified genes were annotated and browsed using tools that we had previously implemented for *E. histolytica* gene discovery [21]. These data are summarized in the following sections.

**Table 1:** Fold-changes for genes differentially expressed in low iron as detected by microarray and quantitative real-time PCR.

Gene description	AmoebaDB ID	Genbank ID	Microarray	qRT-PCR
Cell division control protein 42	EHI_154270	XM_645351	4.5	3.9±1.1
Glutamyl-tRNA synthetase	EHI_155570	XM_650693	3.1	3.3±1.4
Regulator of nonsense transcripts	EHI_110840	XM_649191	3.0	36±19.4
S-adenosylmethionine synthetase	EHI_195110	XM_001913755	2.9	3.3±2.0
Hypothetical protein	EHI_023330	XM_650547	2.6	5.1±1.5
Fe-hydrogenase	EHI_005060	XM_647747	−2.9	−3.1±0.1
Grainin 2	EHI_167310	XM_645265	−3.1	−1.7±0.6
Actobindin	EHI_039020	XM_651745	−3.3	−1.7±0.1
Grainin 2	EHI_111720	XM_001913814	−3.3	−1.8±0.1
Actobindin	EHI_158570	XM_644616	−4.0	−3.2±0.1
Monooxygenase	EHI_009840	XM_652013	−4.4	−2.6±0.3

AmoebaDB ID and Genbank ID refers to the accession number of the gene in AmoebaDB and NCBI GenBank, respectively; Microarray and qRT-PCR refers to fold-change in the low iron condition as compared with the normal iron condition as detected using microarray.

### Genes that are differentially expressed in iron deficiency

Genes that are differentially expressed in iron deficiency might be important for the parasite's adaptation to long-term iron deficiency (Table 2 and Table S2 in File S1). Upregulated transcripts included genes coding for acyl-CoA synthetase, ComEC competence proteins, androgen-inducible gene 1 (AIG1) and NADPH-dependent oxidoreductase (EhNO_2_). Acyl-CoA synthetase (coded for by EHI_153060) is essential enzyme for *de novo* lipid synthesis, fatty acid catabolism, vesicular trafficking, membrane remodeling. It is also involved in the post-translational modification of proteins and the regulation of gene expression [25]. The ComEC competence proteins are putative channels for DNA uptake in bacteria [26]. They contain seven transmembrane domains, a competence (COM) domain and a metallo-β-lactamase domain. The EHI_169340 and EHI_156240 proteins (annotated as “amebic ComEC”) are smaller than the bacterial homologs and do not appear to contain a COM domain (Figure S3 in File S1). Functional activity has not yet been described for amebic ComEC proteins. The ComEC EHI_169340 and EHI_156240 transcripts were upregulated in iron deficiency. After Hb supplementation, the ComEC EHI_169340 transcript declined to normal levels but the ComEC EHI_156240 was still upregulated (Table 2). AIG1 is a member of the GTPase immunity-associated protein family [27], [28]. Three AIG1 transcripts (EHI_115160, EHI_022500, and EHI_195260) were upregulated in iron deficiency (Table 2). These three genes are also upregulated in an *E. histolytica* cell line that produce large liver abscesses in a gerbil model but are not modified in an *E. histolytica* cell line that does not produce abscesses. Hence, Biller et coworkers have suggested that the AIG1 gene is a pathogenicity factor in *E. histolytica* [29]. The EHI_195260 transcript is downregulated in trifluoromethionine-resistant trophozoites [30]. Other members of the AIG gene family (such as EHI_144280 and EHI_144390) are known to be overexpressed in trophozoites during mouse intestinal colonization [31]. EhNO_2_ is involved in redox homeostasis through L-cysteine and iron reduction [32] and was also upregulated in iron deficiency (Table 2).

**Table 2:** Differentially expressed genes relevant to the iron uptake, utilization and storage.

Gene description	AmoebaDB ID	Genebank ID	Iron deficiency	Iron def + Hb	Low iron
**Genes that are preferentially upregulated in iron deficiency**					
Acyl-CoA synthetase	EHI_153060	XM_651318	5.7	5.4	NM
AIG1 family protein	EHI_195260	XM_643194	2.6	NM	NM
AIG1 family protein	EHI_022500	XM_642923	2.3	NM	NM
AIG1 family protein	EHI_115160	XM_644114	2.2	NM	NM
Competence protein ComEC	EHI_156240	XM_647820	6.5	4.1	NM
Competence protein ComEC	EHI_169340	XM_650745	2.4	NM	NM
EhNO2	EHI_045340	XM_648481	2.2	NM	NM
**Genes that are preferentially upregulated after Hb supplementation**					
Acyl-CoA synthetase	EHI_131880	XM_647524	NM	2.2	NM
Alcohol dehydrogenase 3	EHI_160670	XM_001914165	NM	3.7	NM
Hypothetical protein	EHI_058920	XM_646161	2.4	4.2	NM
Hypothetical protein	EHI_009990	XM_651985	NM	4.1	NM
Hypothetical protein	EHI_187790	XM_643995	NM	3.7	NM
Hypothetical protein	EHI_174580	XM_648227	NM	3.7	NM
Hypothetical protein	EHI_075990	XM_649526	NM	3.7	NM
Hypothetical protein	EHI_159670	XM_645153	NM	3.2	NM
Hypothetical protein	EHI_148870	XM_652487	NM	2.6	NM
Hypothetical protein	EHI_112830	XM_651012	NM	2.6	NM
Hypothetical protein	EHI_031640	XM_648447	NM	2.1	NM
Hypothetical protein	EHI_151930	XM_652295	NM	4.5	NM
Hypothetical protein	EHI_169830	XM_650917	NM	3.6	NM
Hypothetical protein	EHI_087110	XM_651036	NM	2.5	NM
Hypothetical protein	EHI_050590	XM_651508	NM	2.4	NM
Protein kinase	EHI_140330	XM_646643	NM	3.7	NM
RIO1 family protein	EHI_170330	XM_645949	NM	4.5	NM
STIRP	EHI_004340	XM_001913561	NM	2.2	NM
**ABC and Major Facilitator Transporters**					
ATP binding cassette	EHI_095820	XM_649804	NM	2.9	NM
ATP binding cassette	EHI_178050	XM_646404	NM	2.0	NM
ATP binding cassette	EHI_178580	XM_001913406	NM	2.2	2.2
P-glycoprotein 5	EHI_175450	XM_644247	NM	3.2	2.4
P-glycoprotein 5	EHI_125030	XM_644884	NM	4.8	2.1
P-glycoprotein 5	EHI_075410	XM_001914252	NM	2.1	NM
Transporter major facilitator	EHI_173950	XM_647419	NM	2.5	NM
**GluRS, SAMS and Monooxygenase**					
Glutamyl-tRNA synthetase	EHI_155570	XM_650693	NM	3.4	3.1
Monooxygenase	EHI_009840	XM_652013	−3.7	−20.6	−4.4
S-adenosylmethionine synthetase	EHI_004920	XM_001913609	−2.3	NM	2.9
S-adenosylmethionine synthetase	EHI_174250	XM_647762	−3.5	NM	2.7
S-adenosylmethionine synthetase	EHI_195110	XM_001913755	−2.9	NM	2.5

AmoebaDB ID and Genbank ID refers to the accession number of the gene in AmoebaDB (http://amoebadb.org/amoeba/) and NCBI GenBank (http://www.ncbi.nlm.nih.gov/genbank/), respectively; iron deficiency refers to the fold changes of expression level in the iron deficiency condition as compared with the normal condition; Iron def + Hb refers to the fold-change of the expression level in iron deficiency following by hemoglobin supplementation as compared with the normal condition; low iron refers to the fold-change of expression level in low iron as compared with the normal condition; Please see methods for definition of iron deficiency, low iron, and normal iron condition. NM: non-modulated.

We observed that a number of transcripts were modulated upon Hb supplementation after iron deficiency (Table 2). However, most of these encoded proteins of unknown function. The genes for alcohol dehydrogenase 3 (EHI_160670), acyl-CoA synthetase (EH_131880), kinase (EHI_140330), serine/threonine-protein kinase RIO1 (EHI_170330) and serine/threonine rich protein (STIRP, EHI_004340) were also upregulated (Table 2). The kinase RIO1 is involved in ribosome biogenesis, whereas STIRP is associated with pathogenicity.

### Genes that are potentially responsible for iron uptake and heme/iron metabolism

Comparison of gene expression in the three different conditions enabled us to highlight factors that are likely to be important for iron or heme uptake and trafficking (Table 2). Six putative transmembrane transporters were upregulated in the iron deficiency + Hb condition; (i) three P-glycoprotein-5 transporters (PgP5, EHI_175450, EHI_125030, EHI_075410) and (ii) three ABC transporters (EHI_095820, EHI_178050, EHI_178580). Furthermore, an MFT family member (EHI_173950) (Table 2) also showed increased transcript levels in the iron deficiency + Hb condition. Transcript levels for EHI_178580, EHI_175450 and EHI_125030 were also upregulated under low-iron conditions (Table 2). The ABC transporter (EHI_095820) had already been identified in a proteomic analysis of mitosomes [33]. P-glycoprotein-5 (EHI_175450 and EHI_125030) shows 45% homology with the ATP-binding cassette LABCG5 protein that is involved in intracellular heme trafficking in the parasite *Leishmania* [34]. Furthermore, P-glycoprotein-5 (EHI_175450) and ABC transporters (EHI_095820, EHI_178580, and EHI_178050) show similarities with the domain architecture of the iroC siderophore exporter in *Salmonella typhi* (Figure S3 in File S1) [35]. P-glycoprotein-5 (EHI_125030) shows homology with the PvdE siderophore pyoverdin exporter in *Pseudomonas aeruginosa* [36] (Figure S3 in File S1). Moreover, MFT (EHI_173950) shows similarities with both MFS1 (the azotochelin siderophore exporter in *Azotobacter vineldii* [37]) and FLVCR1 (a human cytoplasmic heme exporter) [38], [39]. Furthermore, MFT is overexpressed in response to L-cysteine deprivation, which suggests that it is involved in metabolite intake or efflux in *E. histolytica* [40]. Further experiments are necessary to clarify the role of ABC transporters and MFTs in siderophore/hemophore transport in *E. histolytica*.

It is noteworthy that genes relevant for heme metabolism in bacteria were modulated in trophozoites grown under both iron deficiency and low-iron conditions. These include genes coding for three S-adenosylmethionine synthetases (SAMS; EHI_174250, EHI_004920, and EHI_195110), a monooxygenase (EHI_009840) and glutamyl-tRNA synthetase (GluRS, EHI_155570) (Table 2). The SAMS transcripts were downregulated in iron deficiency but normal levels were restored after Hb supplementation (Table 2). S-adenosylmethionine synthetase is responsible for the biosynthesis of S-adenosylmethione (SAM); the latter is necessary in many biosynthetic pathways, including methionine-cysteine conversion [40] and heme biosynthesis [41], [42]. S-adenosylmethionine synthetase is also involved in iron acquisition in plants and it is necessary for the synthesis of mugineic acid (MA) phytosiderophores through a well described pathway [43].

The monooxygenase-encoding transcript was downregulated moderately under low-iron and iron deficiency conditions and dramatically (20-fold) in the iron deficiency + Hb condition (Table 2). The monooxygenase (EHI_009840) presents significant homology (Figure in File S1) with bacterial monooxygenases (such as the heme-degrading monooxygenase (isdG) from *Staphylococcus aureus*). The *E. histolytica* gene coding for glutamyl-tRNA synthetase (EHI_155570) was upregulated under iron deficiency + Hb and low-iron conditions (Table 2). Expression of the amebic GluRS encoding gene did not change during iron starvation but was upregulated by a factor of 3.4 in the iron deficiency + Hb condition. Glutamyl-tRNA synthetase is involved in heme/tetrapyrrole biosynthesis in bacteria and plants.

We used qRT-PCR to further examine expression levels of SAMS, monooxygenase and GluRS transcripts by (Tables 3, S5 and S6 in File S1). The SAMS transcript (EHI_195110) was upregulated under low-iron conditions and was downregulated in iron deficiency. However, Hb supplementation was associated with a return to normal expression levels of SAMS (Tables 3). Glutamyl-tRNA synthetase gene expression was not modulated in the iron-deficient environment but was upregulated under both low-iron and iron deficiency + Hb environments. Moreover, we confirmed that SAMS and GluRS transcripts were modulated only in trophozoites having been exposed to below-normal iron concentrations, since the transcript levels did not vary significantly in trophozoites grown in normal iron medium supplemented with Hb for 2 hrs (qRT-PCR, Table S5 in File S1). Levels of monooxygenase transcript increased by a factor of 1.8 in normal iron medium supplemented with Hb for 2 hours (Table S5 in File S1) and decreased by a factor of 20 in the iron deficiency + Hb condition (Table 2), as observed with transcriptome analyses.

**Table 3:** Fold-changes for genes differentially expressed in low iron condition detected by quantitative real-time PCR.

Gene description	AmoebaDB ID	Genbank ID	Low iron	Low iron + Hb
S-adenosylmethionine synthetase	EHI_195110	XM_001913755	3.3±2.0	1.1±0.1
Glutamyl-tRNA synthetase	EHI_155570	XM_650693	3.3±1.4	1.2±0.5

AmoebaDB ID and Genbank ID refers to the accession number of the gene in AmoebaDB and NCBI GenBank, respectively; low iron refers to the to fold-change in low iron condition compared with normal iron condition detected by quantitative real-time PCR; Low iron + Hb refers to the to fold-change in low iron condition with hemoglobin supplementation compared with normal iron condition detected by quantitative real-time PCR.

To confirm that SAMS, GluRS, and monooxygenase transcript levels were regulated by iron deficiency and that iron from Hb and/or AFC supplementation reestablished their expression, transcript amounts were determined in trophozoites grown in iron-deficient medium for only 24 hrs. In this condition, expression was similar to that observed under low-iron conditions; SAMS and GluRS expression levels were upregulated 2.9- and 2.5-fold, respectively (Table 2 and Table S6 in File S1). However, levels of the monooxygenase transcript were 6.3-fold lower. Transcript expression returned to baseline levels when the iron-deficient medium was replaced by normal medium for 24 hours (Table S6 in File S1). Overall, we confirmed the modulation of SAMS, GluRS, and monooxygenase transcripts by iron deficiency and thus validated the microarray data.

The differential regulation of the genes coding for GluRS and SAMS in *E. histolytica* grown in various iron concentrations may indicate a potential role in heme biosynthesis, given the enzymes' reported role in bacteria, plants, and humans. However, a heme biosynthesis pathway has not yet been described in *E. histolytica.* In a BLAST search for orthologous genes encoding enzymes responsible for the production of components within the heme biosynthesis pathway (from aminolevulinic acid to heme production), we observed that only two genes in *E. histolytica* (the hypothetical proteins EHI_138420 and EHI_095090) present homology with intermediate enzymes. The hypothetical protein EHI_138420 shows homology with uroporphyrin-III C-methyltransferase from *Actinobacillus pleuropneumoniae* (32% similarity), whereas the hypothetical protein EHI_095090 presents homology with ferrochelatase from *Nitrosomonas sp* (27.4% similarity) (Figure S3 in File S1). Ferrochelatase is an enzyme that catalyzes the terminal step in heme biosynthesis (conversion of protoporphyrin IX (PIX) into heme). Thus, further experiments are necessary to characterize the role of the hypothetical protein EHI_138420 and the ferrochelatase-like protein (EHI_095090) in *E. histolytica*.

### Molecular functions that are potentially regulated by changes in iron levels

When considering the functional category, we found that differentially expressed genes belonged to groups related to oxidoreductase activity, sulfur-containing amino acid metabolism, general stress responses, DNA repair, RNA synthesis, cysteine proteinases (CP), and actin cytoskeleton rearrangements. The genes regulated within each of these categories are described below.

#### Oxidoreductase activity

Nineteen genes encoding proteins with oxidoreductase activity were upregulated in the iron deficiency + Hb condition (Table 4). *Entamoeba histolytica* lacks the components for MA siderophore biosynthesis. However, we found that *E. histolytica* aldose reductase shows homology with the deoxymugineic acid synthases from *Zea mays* (maize) and *Oryza sativa* (rice). Furthermore, an aldo-keto reductase from the green algae *Chlorella vulgaris* with aldehyde reductase activity is capable of functioning as a ferric reductase and driving the Fenton reaction. In the presence of Fe^2+^ as an electron donor, hydrogen peroxide is univalently reduced to produce the hydroxyl radical, which can then produce toxic ROS [44]. Three aldose reductase transcripts (EHI_157010, EHI_039190, and EHI_107560) were upregulated 5.0- to 5.6-fold under the iron deficiency + Hb condition only (Table 4). Hence, further work will have to clarify the function of aldose reductase in the siderophores biosynthesis and/or ROS formation in *E. histolytica*.

**Table 4:** Differentially expressed genes relevant to oxidoreductase activity and sulfur-containing amino acid metabolism.

Gene description	AmoebaDB ID	Genbank ID	Iron deficiency	Iron def + Hb	Low iron
**Oxidation-reduction**					
Alcohol dehydrogenase	EHI_125950	XM_645327	NM	−3.9	−3.6
Alcohol dehydrogenase ADH3	EHI_160670	XM_001914165	NM	3.7	NM
Alcohol dehydrogenase ADH2	EHI_150490	XM_647208	NM	2.5	NM
Alcohol dehydrogenase ADH3	EHI_088020	XM_643978	NM	4.2	2.8
Aldehyde-alcohol dehydrogenase ADH2	EHI_160940	XM_650725	NM	2.2	NM
Aldehyde-alcohol dehydrogenase ADH2	EHI_024240	XM_001913618	NM	2.3	NM
Aldose reductase NADPH-dependent oxidoreductase	EHI_157010	XM_001914421	NM	5.6	NM
Aldose reductase NADPH-dependent oxidoreductase	EHI_039190	XM_648674	NM	5.1	NM
Aldose reductase NADPH-dependent oxidoreductase	EHI_107560	XM_001914234	NM	5.0	NM
Fe-hydrogenase	EHI_005060	XM_647747	NM	2.0	−2.9
Fe-S cluster assembly NifU	EHI_049620	XM_650796	NM	2.7	NM
Hydroxylamine reductase	EHI_004600	XM_644914	NM	2.5	NM
Iron sulfur flavoprotein	EHI_022600	XM_643169	−2.9	2.2	NM
Iron sulfur flavoprotein	EHI_067720	XM_643101	−2.6	2.0	NM
Iron sulfur flavoprotein	EHI_022270	XM_644774	−2.3	2.0	NM
Iron sulfur flavoprotein	EHI_103260	XM_001913434	−2.3	NM	NM
Iron sulfur flavoprotein, FprB2	EHI_138480	XM_650038	NM	3.3	2.8
Iron-containing superoxide dismutase (Fe-SOD)	EHI_159160	XM_643735	NM	2.5	NM
Malate dehydrogenase	EHI_014410	XM_001913511	NM	2.4	NM
Malate dehydrogenase	EHI_165350	XM_644664	NM	2.1	NM
NADP-dependent alcohol dehydrogenase	EHI_023110	XM_648415	NM	−2.4	−2.7
Sulfotransferase	EHI_197340	XM_646583	NM	2.0	2.8
**Sulfur-containing amino acid metabolism**					
Cysteine desulfurase NifS	EHI_136380	XM_650165	NM	3.2	NM
Cysteine synthase CS1	EHI_171750	XM_648014	NM	3.8	NM
Cysteine synthase CS2	EHI_160930	XM_643199	NM	3.9	2.5
D 3 phosphoglycerate dehydrogenase PGDH	EHI_060860	XM_647048	−2.0	−2.1	NM
Methionine gamma lyase	EHI_057550	XM_001913898	NM	−2.6	NM
Methionine gamma lyase MGL1	EHI_144610	XM_647004	NM	−2.1	NM
Phosphoglycerate dehydrogenase PSAT	EHI_026360	XM_650291	−2.0	−2.2	NM

For definition of columns please refer to footnote of Table 2.

An iron-containing superoxide dismutase transcript was also identified. Levels of this transcript were not altered in trophozoites grown under iron deficiency or low-iron conditions. Iron-containing superoxide dismutase has been identified as a dimeric enzyme responsible for superoxide radical (O2•^−^) detoxification in *E. histolytica* [45]. The downregulated genes in the iron deficiency condition included four iron sulfur flavoproteins (ISFs) (EHI_022600, EHI_022270, EHI_067720, and EHI_103260; Tables 4 and S2 in File S1). Expression of the ISF EHI_138480 gene expression was not altered by iron deficiency but was upregulated upon Hb supplementation and under low-iron conditions. The ISFs constitute a novel family of proteins that are broadly distributed across distantly related anaerobes. *Trichomonas vaginalis* and *E. histolytica* are the only eukaryotes to possess ISFs [40], [46]. The ISF EHI_067720, EHI_103260, and EHI_138480 transcripts were upregulated during L-cysteine deprivation [40], and the ISF EHI_067720 was downregulated in a mouse model of intestinal amebiasis [31].

Alcohol dehydrogenase transcripts were upregulated in the iron deficiency + Hb condition; they included aldehyde-alcohol dehydrogenase 2 (EHI_024240; a 2.3-fold increase), which known to be involved in the internalization of human transferrin [47] and is regulated by iron [3]. Alcohol dehydrogenase (EHI_125950) and NADP-dependent alcohol dehydrogenase (EHI_023110) transcripts were downregulated in trophozoites grown under low iron concentrations and upon Hb supplementation. The Fe-hydrogenase (EHI_005060) transcript was downregulated 2.9-fold under low iron conditions (Table 4) and upregulated 2-fold after supplementation with Hb. Transcripts such as alcohol dehydrogenase (EHI_088020) and sulfotransferase (EHI_197340) were also differentially expressed in trophozoites cultured under low iron conditions (Table 4).

#### Sulfur-containing amino acid metabolism

Cysteine synthase CS1 (EHI_171750) and CS2 (EHI_190630) transcripts and cysteine desulfurase NifS (EHI_136380) transcripts were upregulated in the iron deficiency + Hb condition (Table 4). Together with the changes observed for SAMS (Table 2), this upregulation suggests that cysteine metabolism is activated and forms a key part of the metabolism of sulfur-containing amino acids in *E. histolytica* [48], [49]. Upregulation of the NifS transcript also suggests that Fe-S cluster synthesis is activated in the presence of Hb as an iron source (Table 4). In contrast, serine metabolism appears to be downregulated, since transcripts of genes coding for D-3-phosphoglycerate dehydrogenase (PGDH, EHI_060860), methionine γ-lyase 1 (MGL1, EHI_144610), and phosphoserine aminotransferase (PSAT, EHI_026360) were downregulated in iron deficiency. The PGDH and PSAT transcripts were still downregulated after Hb supplementation.

#### Stress responses, DNA repair and RNA synthesis

A number of transcripts involved in stress responses were upregulated upon Hb replenishment, including heat shock proteins (HSPs) such as Hsp101 and ClpB (Table 5) and the chaperone Hsp90. Hsp101 and ClpB transcripts were also upregulated in the low-iron condition but not in the iron deficiency condition. Upon Hb replenishment, Hsp20 was downregulated and Hsp70A2 was upregulated. Thus, in an iron-deficient environment, the sudden supply of heme might induce a stress response in *E. histolytica*. Gene transcripts involved in DNA/RNA synthesis and DNA repair (such as the double strand break repair protein MRE11, DEAD/DEAH box helicase and the regulators of nonsense transcripts) were modulated by Hb supplementation (Table 5). Expression of three regulators of nonsense transcripts was observed in amoebae grown in the low-iron condition (Table 5) and upon supplementation with Hb. The regulators of nonsense transcripts present homology with the product of the human *Upf1* gene (regulator of nonsense transcripts 1, a RNA helicase that detects mRNA containing premature stop codons).

**Table 5:** Differentially expressed genes relevant to heat shock stress, DNA repair, and RNA synthesis.

Gene description	AmoebaDB ID	Genbank ID	Iron deficiency	Iron def + Hb	Low iron
**Stress response**					
Chaperone clpB	EHI_090840	XM_001914528	NM	2.9	3.9
Chaperone clpB	EHI_155060	XM_001914269	NM	2.1	3.5
Chaperone clpB	EHI_094680	XM_001914511	NM	NM	2.7
Heat shock protein 101	EHI_076480	XM_001914516	NM	NM	3.3
Heat shock protein 101	EHI_013550	XM_001914488	NM	NM	2.8
Heat shock protein 101	EHI_156560	XM_001914239	NM	2.2	4.1
Heat shock protein 101	EHI_183680	XM_001914603	NM	2.1	3.9
Heat shock protein 101	EHI_178230	XM_001914365	NM	NM	3.8
Heat shock protein 101	EHI_094470	XM_001914472	NM	NM	2.5
Heat shock protein 90	EHI_196940	XM_648040	NM	2.0	NM
Heat shock protein Hsp20	EHI_125830	XM_651403	NM	−2.1	NM
Heat shock protein70 (hsp70A2)	EHI_015390	XM_001913629	NM	2.1	NM
**DNA repair**					
DNA directed RNA polymerase II subunit	EHI_056690	XM_643999	NM	2.1	NM
DNA directed RNA polymerase III subunit	EHI_050830	XM_651537	NM	2.0	NM
Double strand break repair protein MRE11	EHI_125910	XM_651393	NM	3.9	NM
**RNA synthesis**					
DEAD box ATP dependent RNA helicase 42	EHI_197990	XM_001914267	NM	2.0	NM
DEAD/DEAH box helicase	EHI_131080	XM_650428	NM	3.7	NM
Regulator of nonsense transcripts	EHI_070810	XM_649317	NM	3.7	3.8
Regulator of nonsense transcripts	EHI_110840	XM_649191	NM	2.7	3.0
Regulator of nonsense transcripts	EHI_035550	XM_651038	NM	2.0	NM
Regulator of nonsense transcripts	EHI_193520	XM_646961	NM	NM	2.2
RNA binding protein	EHI_151990	XM_652289	NM	2.2	NM

For definition of columns please refer to footnote of Table 2.

#### Cysteine proteinases and actin-related cytoskeleton

It has already been suggested that iron-limited conditions regulate cysteine proteinase levels in *E. histolytica* [50]. In the present analysis, we identified three annotated cysteine proteinases (CP-A4, CP-A5, and CP-A7) and the putative cysteine proteinase EHI_010850 (XP_001914429). Upon Hb supplementation, expression of CP-A5, CP-A7, and EHI_010850 was upregulated 2.4-, 4.5-, and 4.5-fold, respectively. Furthermore, CP-A4 was upregulated 3.0-fold in iron-starved trophozoites (Table 6). Transcripts encoding proteins involved in actin cytoskeleton organization (such as actobindin (EHI_158570, EHI_039020), cofilin (EHI_186840), and ARP 2/3 complex subunits (EHI_199690, EHI _045000)) (Table 6) were downregulated only in low-iron conditions. Reorganization of the actin cytoskeleton may thus be correlated with the previously reported loss of parasite adherence under low-iron conditions [5].

**Table 6:** Differentially expressed genes relevant to other notable molecular functions.

Gene description	AmoebaDB ID	Genbank ID	Iron deficiency	Iron def + Hb	Low iron
**Cysteine Proteinases**					
Cysteine proteinase	EHI_010850	XM_001914394	NM	4.5	NM
Cysteine proteinase (CP-A7)	EHI_039610	XM_643904	NM	4.5	NM
Cysteine proteinase (CP-A4)	EHI_050570	XM_651510	3.0	NM	NM
Cysteine proteinase (CP-A5)	EHI_168240	XM_645845	NM	2.4	NM
**Actin cytoskeleton organization**					
Actobindin	EHI_158570	XM_644616	NM	NM	−4.0
Actin binding protein cofilin/tropomyosin	EHI_186840	XM_651345	NM	NM	−2.4
Actin related protein 2/3 complex subunit 1A	EHI_045000	XM_652168	NM	NM	−2.0
Actobindin	EHI_039020	XM_651745	NM	NM	−3.3
ARP2/3 complex 34 kDa subunit	EHI_199690	XM_645477	NM	NM	−2.0
**Other amoebic transcripts**					
Acetyltransferase	EHI_039180	XM_001913968	NM	2.0	NM
Acetyltransferase	EHI_167080	XM_649690	NM	−2.1	NM
Aspartate ammonia lyase	EHI_082270	XM_001913840	NM	−2.3	−2.3
Aspartate ammonia lyase	EHI_150390	XM_650734	NM	−2.5	−2.0
Calmodulin	EHI_010020	XM_651988	NM	−2.0	−3.0
Grainin 1	EHI_167300	XM_645280	NM	NM	−2.8
Grainin 2	EHI_111720	XM_001913814	NM	NM	−3.3
Grainin 2	EHI_167310	XM_645265	NM	NM	−3.1
Molybdenum cofactor sulfurase	EHI_194600	XM_649637	NM	−2.1	NM
N system amino acid transporter 1	EHI_050900	XM_649086	NM	2.4	NM
WD domain containing protein	EHI_126260	XM_647361	NM	−2.0	NM

For definition of columns please refer to footnote of Table 2.

Overall, the results of our microarray analysis provide a new vision of iron-related functions in *E. histolytica*. There are clear similarities with bacterial iron-related pathways. Furthermore, our work is the first to highlight candidates for iron uptake and utilization studies *in E. histolytica.*

### Identification of IRE-like sequences

By taking advantage of existing bioinformatics approaches, we screened *E. histolytica*'s entire genome for post-transcriptional regulatory elements controlling cellular iron homeostasis. In eukaryotes, key proteins involved in iron transport and storage (e.g., TfR1 and ferroportin) are regulated post-transcriptionally by *cis* elements present in their mRNA. These IREs are stem-loop structures containing the canonical sequences required to bind IRPs. Iron-responsive elements have been found in both the 5′ and 3′ untranslated regions (UTRs) of mRNAs and also in regions coding for proteins involved in iron transport and storage [51]–[53]. We used the SIREs web server to identify potential IRE structures in the *E. histolytica* transcripts identified by the microarray analysis [24]. We analyzed the 5′-UTR, 3′-UTR and coding mRNA sequences extracted from the *E. histolytica* genome. The results were then categorized into SIREs' three stringency levels and the presence of putative IRE-like stem-loop structures was determined by inspection of the SIREs output files. In the whole-genome analysis, we identified 550 transcripts containing IRE structures. Of these transcripts, 173 had high- or medium-stringency IREs, which were more present in coding regions than in 5′- and 3′-UTRs. Eighteen transcripts with putative IREs appeared to be significantly regulated in our microarray analyses (Table 7). Five of the latter had IRE-like structures with high and medium stringency levels. The proportion of differentially expressed genes with IRE-containing transcripts is similar to that in the genome as a whole. Furthermore, stem-loop IRE-like structures were identified in transcripts that were upregulated in iron-starved parasites or upon Hb replenishment (e.g., MFT, DEAD/DEAH helicase, AIP family members, and acyl-CoA synthetase) (Table 7). Stem-loop folds were also identified in transcripts that were not modulated (according to the microarray analysis) by iron starvation or Hb replenishment (e.g. thioredoxin and CP-A8). Thus, the presence of IRE-like structures in these mRNAs may control translation during changes in intracellular iron levels.

**Table 7:** IRE-like sequences in differentially expressed genes.

Gene description	AmoebaDB ID	Genbank ID	Iron deficiency	Iron def + Hb	Low iron	RNA	IRE quality	Stem-loop
DEAD/DEAH box helicase	EHI_131080	XM_650428	NM	3.7	NM	CD	H	-
STIR	EHI_025700	XM_644280	NM	2.3	NM	CD	H	-
Hypothetical protein	EHI_092110	XM_649471	NM	NM	−2.4	5′ UTR	H	-
Transporter major facilitator	EHI_173950	XM_647419	NM	2.5	1.6	CD	M	YES
Calmodulin	EHI_023500	XM_650529	NM	−1.4	−2.0	5′ UTR	M	-
Proliferating cell nuclear antigen	EHI_128450	XM_646418	NM	4.2	NM	CD	L	YES
Leucine rich repeat / protein phosphatase 2C	EHI_137760	XM_648955	NM	3.9	NM	CD	L	-
Cysteine synthase A	EHI_160930	XM_643199	NM	3.9	2.5	CD	L	YES
Hypothetical protein	EHI_197520	XM_646601	NM	2.8	NM	CD	L	YES
DEAD/DEAH box helicase	EHI_119920	XM_644837	NM	2.0	NM	CD	L	YES
AIG1 family protein	EHI_022500	XM_642923	2.5	NM	NM	5′ UTR	L	YES
AIG1 family protein	EHI_195260	XM_643194	2.3	NM	NM	5′ UTR	L	YES
AIG1 family protein	EHI_115160	XM_644114	2.2	NM	NM	5′ UTR	L	YES
Acyl-CoA synthetase	EHI_131880	XM_647524	NM	2.2	NM	5′ UTR	L	YES
ARP2/3 complex 20 kDa subunit	EHI_152660	XM_643475	NM	−1.5	−1.8	CD	L	-
Threonine dehydratase	EHI_049910	XM_652079	NM	−1.7	−1.9	CD	L	YES
Hypothetical protein	EHI_053140	XM_001913728	NM	NM	−2.2	CD	L	-
Amino acid transporter	EHI_072120	XM_648362	NM	−2.0	NM	CD	L	-

NM: non-modulated; CD: coding region; 5′UTR: 5′ untranslated region; H: high stringency; M: medium stringency, and L: low stringency.

## Discussion

*Entamoeba histolytica* must adapt its metabolism as a function of the changing iron concentrations encountered during host infection. It also needs to acquire iron from host sources. In the present study, we searched for genes whose *in vitro* expression level was modulated by differing iron conditions (i.e. cues for iron uptake, utilization and storage in *E. histolytica*). *Entamoeba histolytica* has hemophore-like proteins [14] and receptors for transferrin and lactoferrin [13]. The mechanisms of heme-hemophore and iron-siderophore uptake and the subsequent iron utilization and storage have not been described. In our transcriptome analysis, we identified several genes that encode factors with transporter functions (such as ABC proteins, P-glycoprotein 5 transporters and MFTs). Since homologs of these transporter families are involved in siderophore export/import in bacteria and heme export in humans [35]–[38], they are likely to have a role in siderophore or hemophore export in *E. histolytica* (although this remains to be explored).

In eukaryotes, iron can be recovered from heme via the action of the endoplasmic reticulum heme oxygenase-1 (HO-1) [54]. A gene encoding a monooxygenase was strongly downregulated (20-fold) in iron-deficient trophozoites supplemented with Hb. In bacteria and human cells, monooxygenase is responsible for heme-degradation. Hence, the repression of monooxygenase in the iron deficiency + Hb condition may be required to avoid the production of ROS and Fe^2+^ via heme catabolism [55]. The identification of a monooxygenase protein in *E. histolytica* is noteworthy because the parasite may be able to recover iron from heme that is directly internalized or degraded after erythrophagocytosis.

We found that GluRS and SAMS transcripts were modulated by changes in the iron concentration. These enzymes are involved in several metabolic pathways, including heme biosynthesis [41], [42]. The first step in heme biosynthesis is the production of 5-aminolevulinic acid (ALA) via two unrelated pathways: the C_5_-pathway and the Shemin pathway. In the C_5_-pathway, glutamate is metabolized to glutamyl-t-RNA, L-glutamate-1-semialdehyde and then ALA through the action of GluRS, glutamyl t-RNA reductase and glutamate-1-semialdehyde aminotransferase, respectively. In the Shemin pathway, ALA is produced from the condensation of glycine and succinyl co-enzyme A [56], [57]. The condensation of eight molecules of ALA is necessary to form uroporphyrinogen III, which is then converted to protoporphyrin IX. In a reaction catalyzed by ferrochelatase, protoporphyrin IX can incorporate Fe^2+^ to produce heme [57]. S-adenosylmethionine synthetases are involved in this complex pathway by producing SAM (an essential cosubstrate for many enzymatic reactions, including the final steps of heme synthesis) [42]. For instance, a mutation in SAMS in *Rhodobacter sphaeroides* leads to the accumulation of the intermediate coproporphyrin III [42]. In parasites such as *Leishmania* and *Crithidia*, some (but not all) of the enzymatic machinery required for *de novo* heme synthesis is present [58]; genes in the genomes of symbiotic bacteria complement the absence of full heme synthesis in these parasites. Although the heme biosynthesis pathways in *E. histolytica* have not yet been characterized, the presence of SAMS, GluRS, and a hypothetical (EHI_095090) non-conserved ferrochelatase homolog suggests that iron-related biosynthetic pathways merit further exploration. In higher eukaryotes, heme and Fe-S cluster biosynthesis occurs in the mitochondria. However, heme biosynthesis also occurs in bacteria, which lack mitochondria. *E. histolytica* lacks mitochondria but does have mitosomes. Nevertheless, there is currently no evidence to suggest that amebic mitosomes are involved in heme biosynthesis.

**Figure 1:**
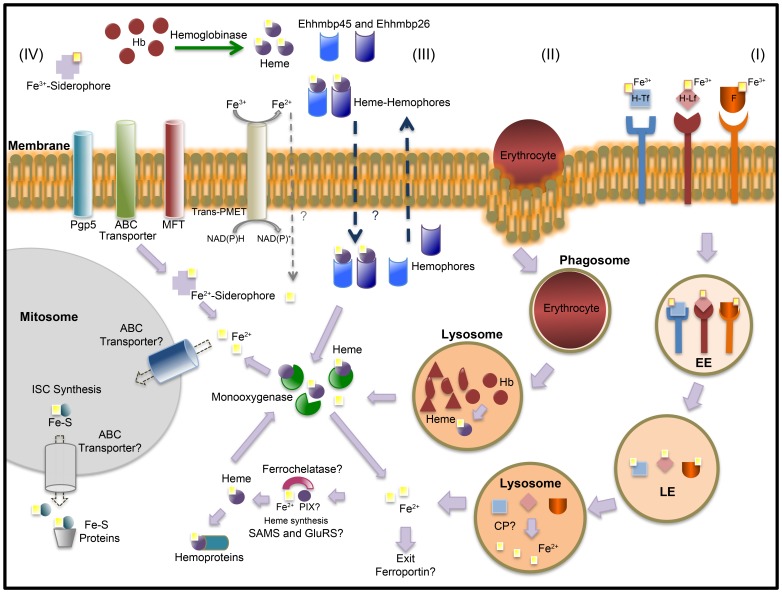
A model summarizing our hypothesis for iron uptake and metabolism in *Entamoeba histolytica*. The pathways described in (I), (II), (III), and (IV) refer to potential routes for iron entry into cells. (I) Trophozoites are able to obtain iron from host proteins such as holotransferrin (H-Tf), hololactoferrin (H-Lf), ferritin (F), and Hb that bind to cell surface proteins. The complexes are internalized in clathrin-coated vesicles (for ferritin and holotransferrin) or caveolin-like coated vesicles (for hololactoferrin). The ligand-receptor complexes dissociate in the endosomes (early endosome; EE and late endosome; LE) and the iron bound to proteins is released in the lysosomes. It is not clear whether iron transporter proteins are degraded by cysteine proteinases (as has been suggested by López-Soto et al [13]) or whether these transporters and cognate receptors are shuttled to the plasma membrane for reuse. (II) *E. histolytica* also acquires iron from Hb via heme internalization during the phagocytosis of human erythrocytes, which are degraded by hemolysin and phospholipases. It has been proposed that hemoglobinases and cysteine proteinases degrade Hb [13]. The monooxygenase heme oxygenase degrades heme and releases iron; we suggest that a monooxygenase (EHI_009840) could be responsible for heme degradation in *E. histolytica.* (III) Heme can also be scavenged by secreted hemophores such as hemoglobin-binding proteins 45 and 26 (Ehhmbp 45 and Ehhmbp 26), which are able to bind Hb and heme [14]. However, the mechanisms of hemophore export and heme-hemophore complex uptake are unknown (blue dashed arrows). An MFT (EHI_173950) and an ABC transporter (EHI_178050) may be involved in heme-hemophore uptake in *E. histolytica*. Once heme has entered the cell, it can be degraded by monooxygenase. (IV) Iron may be taken up by siderophores, since the ABC transporters, P-glycoprotein 5 transporters and the MFT family share homology with bacteria siderophore export systems. Lastly, a trans-plasma membrane electron transport (trans-PMET) system capable of transferring electrons from cytosolic electron donors to non-permeable electron acceptors may have an important role in iron reduction and acquisition (gray dashed arrow) [61]. The above-listed mechanisms deliver iron to cells. The iron could then be used (for example) in iron-sulfur cluster (ISC) biosynthesis in the mitosomes [16]. Heme synthesis has not been described in *E. histolytica*. Both GluRS and SAMS may be key sensors of iron status in this pathway. Ferroportin has not yet been identified in *E. histolytica*.

On the basis of our current knowledge of iron uptake and metabolism in *E. histolytica*, we propose the following hypotheses: (i) *E. histolytica* is able to obtain iron and heme via phagocytosis and directly from host proteins through import mediated by hemophores [14] and siderophores; (ii) transporter and trafficking proteins (e.g., P-glycoprotein 5 proteins, ABC transporters and MFTs) are involved in the internalization and salvaging of iron/heme, as observed for *Leishmania* [34], [58]; (iii) within the cell, iron ions may be released from these complexes through the action of monooxygenase and cysteine proteinases; and (iv) *E. histolytica* maintains a balance between free and protein-bound iron ions using Fe-S clusters. Experimental validation of these hypotheses is now required to confirm the roles of these factors in iron uptake and metabolism in *E. histolytica.* Our working hypothesis is summarized in Figure 1.

Putative IREs are present in prokaryotes and protozoans (e.g., *Plasmodium falciparum* [59] and *Trichomonas vaginalis*). For example, IRE-like structures are present in the mRNA for TVCP4 cysteine proteinase in *Trichomonas vaginalis* [53]. Furthermore, the α-actinin 3 protein (TvACTN3) is able to bind to this IRE-like structure and may be involved in the post-translational mechanism for iron regulation [60]. The amebic cysteine proteinases CP-A7, CP-A5, and CP-A4 were modulated in iron-starved amoebae supplemented with Hb. However, we did not identify IRE-like structures in these mRNAs in a SIREs screen. In contrast, we identified IRE-like structures in amebic STIRP, helicase, MFT, AIG1 and acyl-CoA synthetase mRNAs. Interesting, it has been suggested that the AIG1 genes are *E. histolytica* pathogenicity factors [29]. Iron-regulatory protein homologs that work with IRE elements have yet to be identified in *E. histolytica*. Thus, active IRE-like structures and α-actinin's role in iron regulation in *E. histolytica* have yet to be functionally confirmed.

In summary, we identified candidates for subsequent functional studies of iron/heme uptake, trafficking, and utilization in *E. histolytica*. These included ABC transporters, MFTs, GluRS, SAMS, a monooxygenase, and a ferrochelatase homolog. The regulation of these proteins in presence of ferritin (which is highly abundant in the liver) remains to be investigated.

## Supporting Information

File S1 **Supporting information.** Figure S1, Growth of *Entamoeba histolytica* trophozoites under normal iron conditions (▴) and in iron-deficient medium (▪). Trophozoites were counted in a Neubauer chamber every 24 hrs for 5 days. The cell count corresponds to the mean of three observations. Figure S2, A Venn diagram of genes differentially expressed in three conditions. Upper panel: upregulated genes. Lower panel: downregulated genes. A total of 224 transcripts were significantly modulated in one or more of the three conditions. In iron deficiency, 9 transcripts were upregulated and 11 were downregulated. In iron deficiency + Hb, 107 transcripts were upregulated and 50 were downregulated. Under low-iron conditions, 34 transcripts were upregulated and 46 were downregulated. Figure S3, Alignments of selected *E. histolytica* genes. A) amoebic ComEC orthologs (EHI_169340 and EHI_156240) and ComEC from *Bacillus licheniformis* (WP_003183688.1, 20% similarity), B) amebic P-glycoprotein-5 (EHI_125030) and PvdE from *Pseudomonas aeruginosa* (YP_002440490.1, 33% similarity), C) amoebic MFT (EHI_173950) and MFS1 exporter from *Azotobacter vineldii* (YP_002797373.1, 33% similarity), D) amoebic MFT (EHI_173950) and human FLVCR1 (NP_054772.1), E) amoebic hypothetical protein (EHI_009840) and heme-degrading monooxygenase (isdG) from *Staphylococcus aureus* (NP_645835.1, 38% similarity), F) amoebic hypothetical protein (EHI_095090) and ferrochelatase from *Nitrosomonas sp.* (YP_004294842.1, 27% similarity), and G) amoebic hypothetical protein (EHI_138420) and uroporphyrin-III C-methyltransferase from *Actinobacillus pleuropneumoniae* (WP_005597783.1, 32% similarity). Residues with 100% and 80% homology are highlight in gray and black, respectively. The comparisons were performed using the CLUSTALW alignment tool from the WebExPASy Molecular Biology Server (http://ca.expasy.org). Table S1, Quantification of iron in the TYI-S-33 and TYI-S-33ΔFe medium. *Footnote*: The ferrozine method described in the Material and Methods section was used to quantify iron in the TYI-S-33 medium, incomplete TYI-S-33ΔFe medium (no supplementation with AFC, vitamins and serum) and TYI-S-33ΔFe complete (no supplementation with AFC but supplemented with vitamins and serum) medium. Serum accounts for 55.5 µM iron, i.e. the difference between complete and incomplete media. Peptone accounts for 39.7 µM iron, i.e. the level determined in the incomplete TYI-S-33ΔFe medium. AFC accounts for 78 µM iron, i.e. the difference between the complete TYI-S-33 and complete TYI-S-33ΔFe media. AFC: ammonium ferric citrate; Hb: hemoglobin. Values correspond to the mean of experiments performed in triplicate. Table S2, Differentially expressed genes in iron deficiency. *Footnote*: FC: fold-change; BY: the false discovery rate according to Benjamini and Yekutieli multiple testing; rawp: the unadjusted *P*-value. Table S3, Differentially expressed genes in iron deficiency with Hb supplementation. *Footnote*: FC: fold-change; BY: the false discovery rate according to Benjamini and Yekutieli multiple testing; rawp: the unadjusted *P*-value. Table S4, Differentially expressed genes under low-iron conditions. *Footnote*: FC: fold-change; BY: the false discovery rate according to Benjamini and Yekutieli multiple testing; rawp: the unadjusted *P*-value. Table S5, Fold-changes for genes differentially expressed in normal medium + Hb for 2 hours. *Footnote*: The AmoebaDB ID and GenBank ID numbers refer to the gene's accession number in AmoebaDB and NCBI GenBank, respectively; “Normal iron + Hb for 2 h” refers to the fold-change in expression in TYI-S-33 medium supplemented with Hb for 2 hours, compared with the normal iron condition (as detected by quantitative real-time PCRs). Table S6, Fold-changes for genes differentially expressed in iron deficiency for 24 hours. *Footnote*: The AmoebaDB ID and GenBank ID numbers refer to the gene's accession number in AmoebaDB and NCBI GenBank, respectively; “Normal iron to iron deficiency for 24 h” refers to the fold-change in expression in TYI-S-33 medium without AFC supplementation for 24 hours, when compared with the normal iron condition (as detected by quantitative real-time PCRs). The trophozoites incubated in iron-deficient medium for 24 hours were recovered and incubated for an additional 24 hours in normal iron medium (“iron deficiency 24 h to normal iron 24 h”). Gene expression was detected using quantitative real-time PCRs. Table S7, List of primers used for real time-PCRs. *Footnote*: Position: the relative nucleotide position of the primer's 5′ end, where 0 refers to the first nucleotide of the start codon; Sequence: sequence of the primer from 5′ to 3′. Note that the reverse primer's sequence is reversed and complemented.
(DOC)
