# Supplementary material for: Gene Expression Profiling in Entamoeba histolytica Identifies Key Components in Iron Uptake and Metabolism
Source: PLoS One. 2014 Sep 11;9(9):e107102. doi: 10.1371/journal.pone.0107102 (PMC4161402; doi:10.1371/journal.pone.0107102)
Supplement: File S1 — Supporting information. Figure S1, Growth of Entamoeba histolytica trophozoites under normal iron conditions (▴) and in iron-deficient medium (▪). Trophozoites were counted in a Neubauer chamber every 24 hrs for 5 days. The cell count corresponds to the mean of three observations. Figure S2, A Venn diagram of genes differentially expressed in three conditions. Upper panel: upregulated genes. Lower panel: downregulated genes. A total of 224 transcripts were significantly modulated in one or more of the three conditions. In iron deficiency, 9 transcripts were upregulated and 11 were downregulated. In iron deficiency + Hb, 107 transcripts were upregulated and 50 were downregulated. Under low-iron conditions, 34 transcripts were upregulated and 46 were downregulated. Figure S3, Alignments of selected E. histolytica genes. A) amoebic ComEC orthologs (EHI_169340 and EHI_156240) and ComEC from Bacillus licheniformis (WP_003183688.1, 20% similarity), B) amebic P-glycoprotein-5 (EHI_125030) and PvdE from Pseudomonas aeruginosa (YP_002440490.1, 33% similarity), C) amoebic MFT (EHI_173950) and MFS1 exporter from Azotobacter vineldii (YP_002797373.1, 33% similarity), D) amoebic MFT (EHI_173950) and human FLVCR1 (NP_054772.1), E) amoebic hypothetical protein (EHI_009840) and heme-degrading monooxygenase (isdG) from Staphylococcus aureus (NP_645835.1, 38% similarity), F) amoebic hypothetical protein (EHI_095090) and ferrochelatase from Nitrosomonas sp. (YP_004294842.1, 27% similarity), and G) amoebic hypothetical protein (EHI_138420) and uroporphyrin-III C-methyltransferase from Actinobacillus pleuropneumoniae (WP_005597783.1, 32% similarity). Residues with 100% and 80% homology are highlight in gray and black, respectively. The comparisons were performed using the CLUSTALW alignment tool from the WebExPASy Molecular Biology Server (http://ca.expasy.org). Table S1, Quantification of iron in the TYI-S-33 and TYI-S-33ΔFe medium. Footnote: The ferrozine method described in the Mater [file pone.0107102.s001.doc]

Supplementary materials of

manuscript titled

**Gene expression profiling in *Entamoeba histolytica* identifies key components in iron uptake and metabolism**

by

Nora Adriana Hernández-Cuevas *et al.*

*List of supplementary figures*

**Figure S1**. Growth of *Entamoeba histolytica* trophozoites under normal iron conditions and in iron deficiency medium.

**Figure S2**. A Venn diagram of genes differentially expressed in three conditions.

**Figure S3.** Alignments of selected *E. histolytica* genes.

*List of supplementary tables*

**Table S1.** Quantification of iron in TYI-S-33 and TYI-S-33ΔFe medium.

**Table S2.** Differentially expressed genes in iron deficiency condition.

**Table S3.** Differentially expressed genes in iron starvation deficiency with Hb supplementation.

**Table S4.** Differentially expressed genes in low iron conditions.

**Table S5**. Fold-changes for genes differentially expressed in normal medium + Hb for 2 hours.

**Table S6.** Fold-changes for genes differentially expressed in in iron deficiency for 24 hours.

**Table S7.** List of primers used for real-time PCRs.

**Figure S1**

**
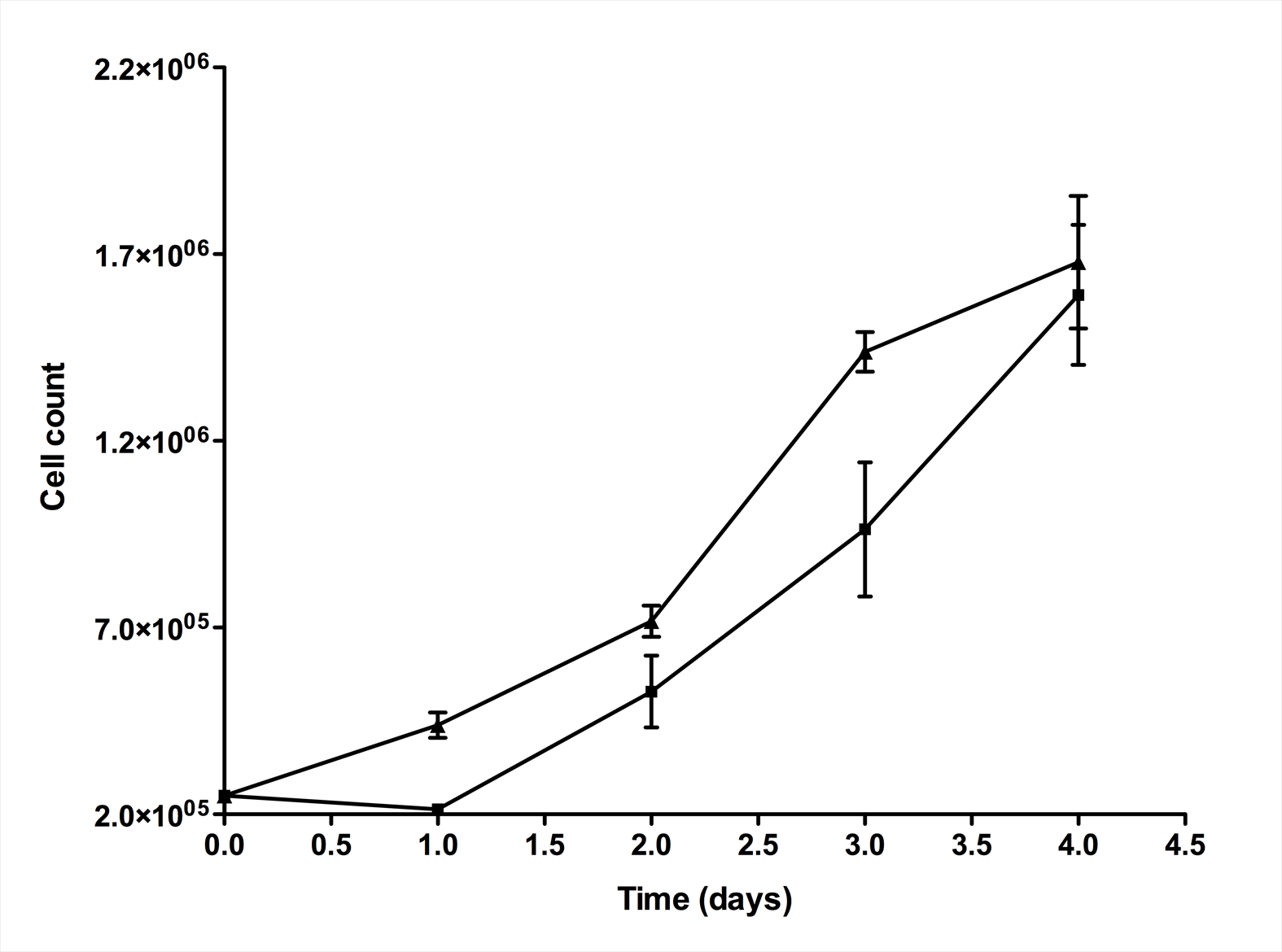
**

**Figure S1. Growth of *Entamoeba histolytica* trophozoites under normal iron conditions (▲) and in iron-deficient medium (■).** Trophozoites were counted in a Neubauer chamber every 24 hrs for 5 days. The cell count corresponds to the mean of three observations.

**Figure S2**

**
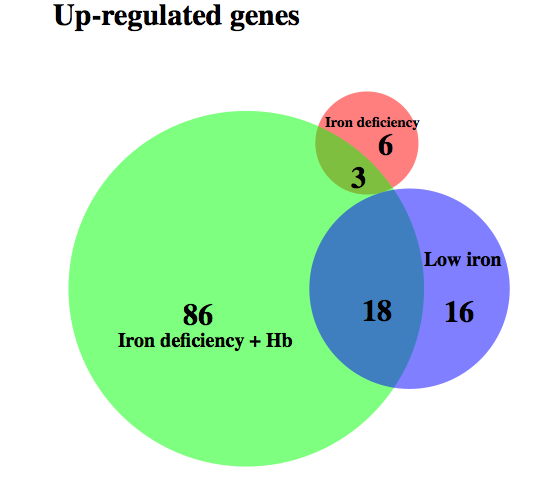
**

**
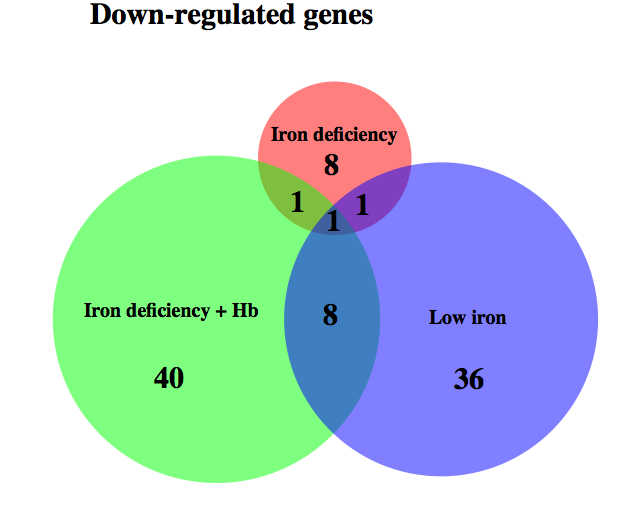
**

**Figure S2**. **A** **Venn** **diagram of genes differentially expressed in three conditions.** Upper panel: upregulated genes. Lower panel: downregulated genes. A total of 224 transcripts were significantly modulated in one or more of the three conditions. In iron deficiency, 9 transcripts were upregulated and 11 were downregulated. In iron deficiency + Hb, 107 transcripts were upregulated and 50 were downregulated. Under low-iron conditions, 34 transcripts were upregulated and 46 were downregulated.

**Figure S3**


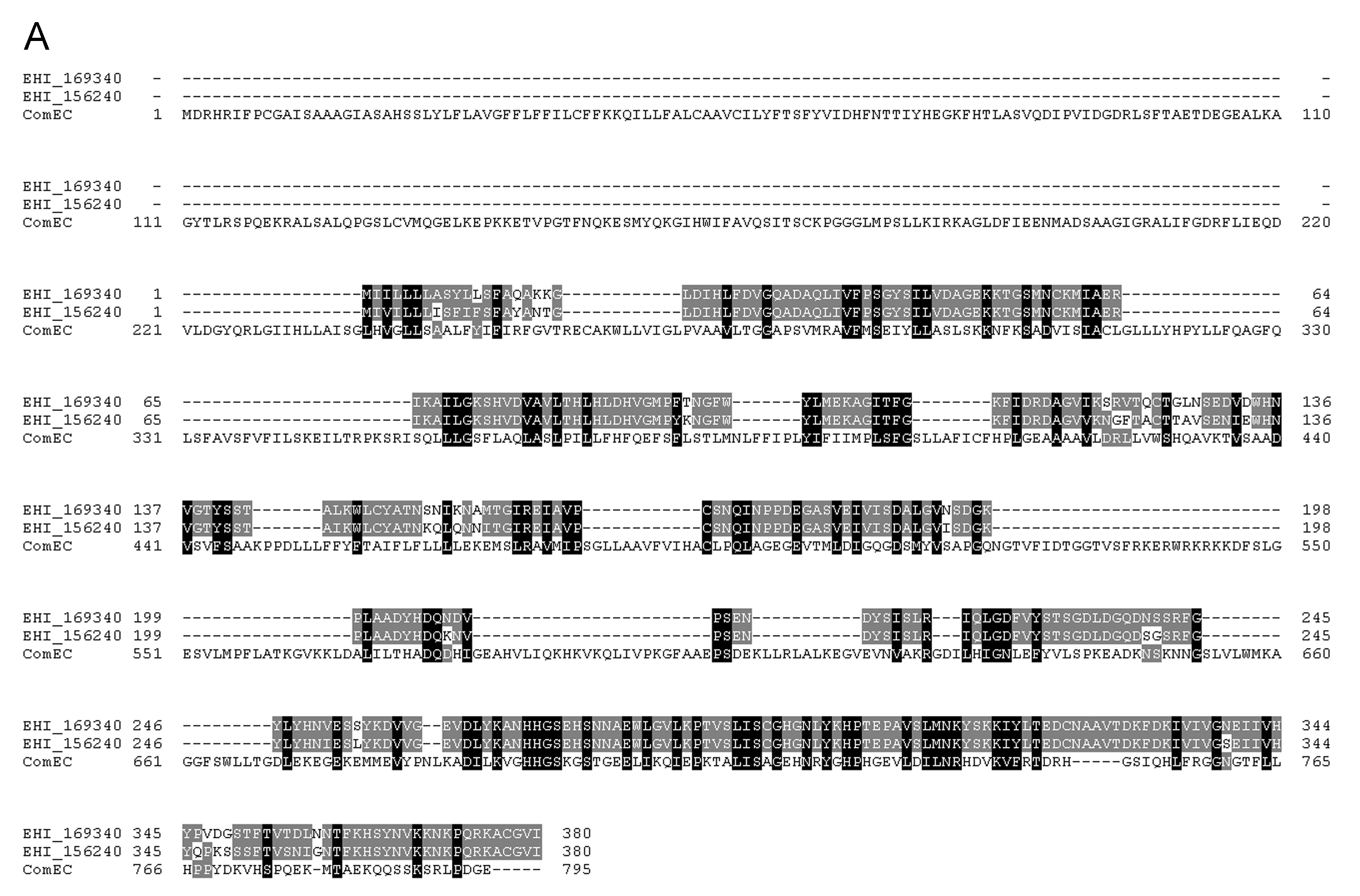


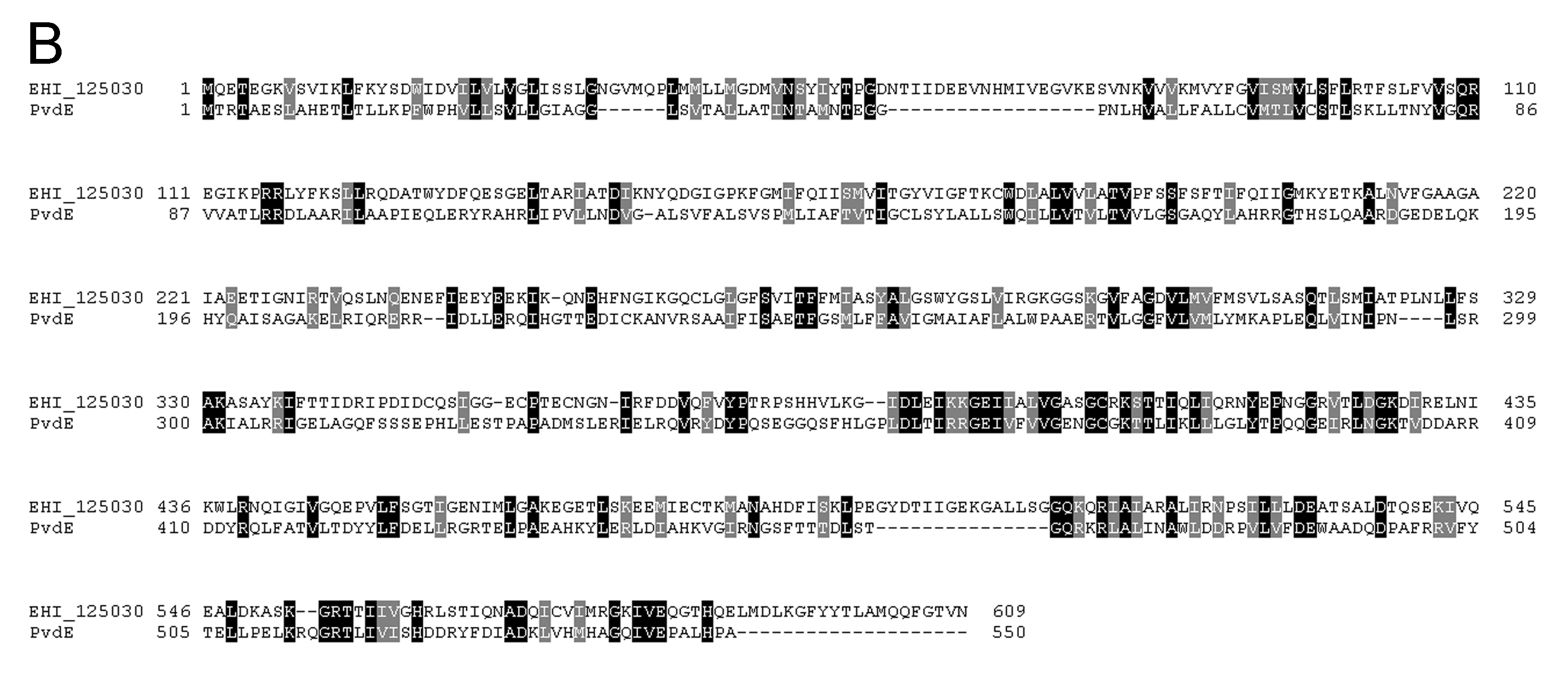


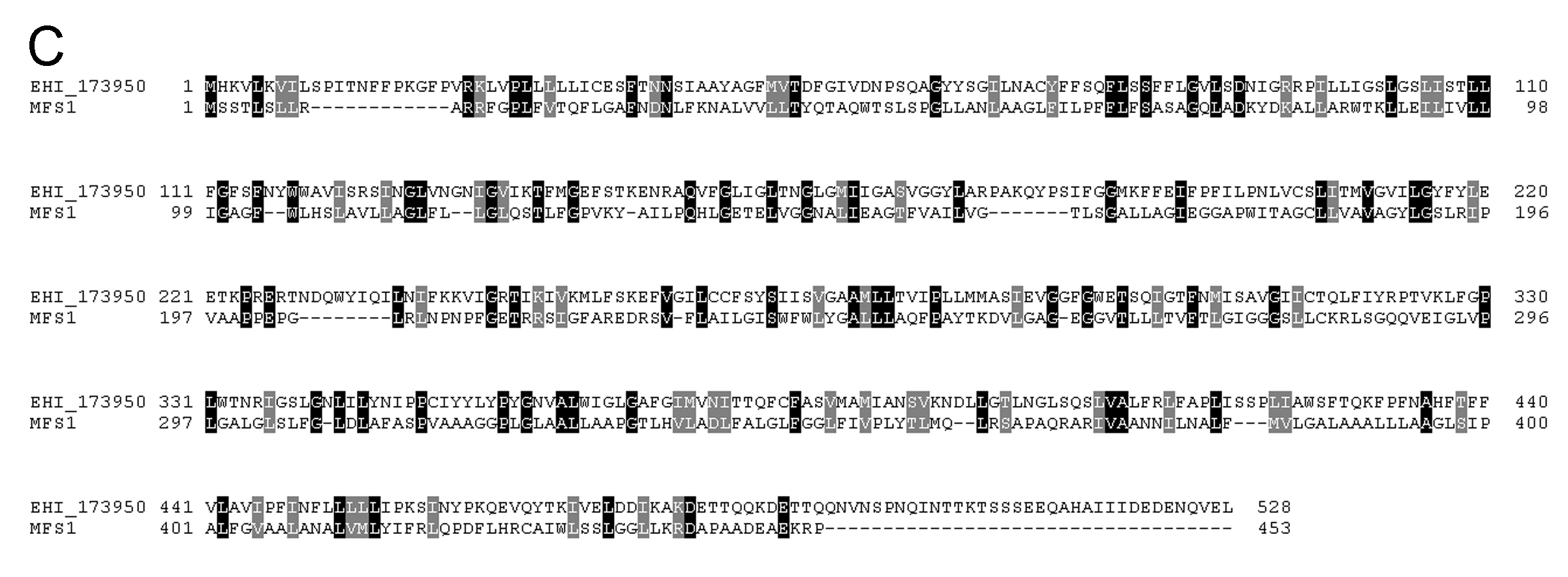

**Figure S3**. **Alignments of selected *E. histolytica* genes. (cont’)**


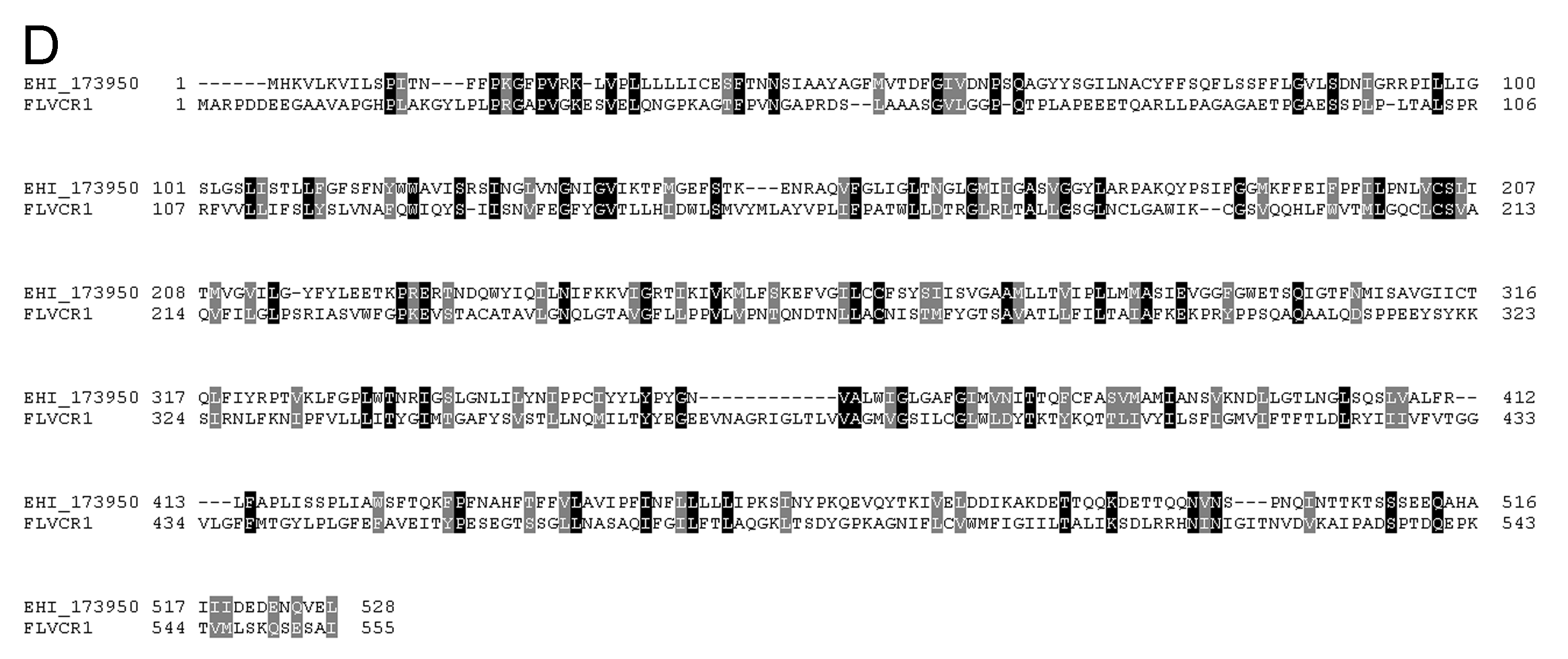


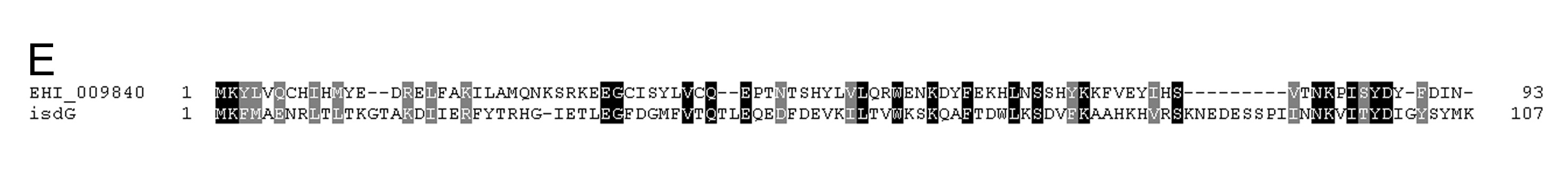


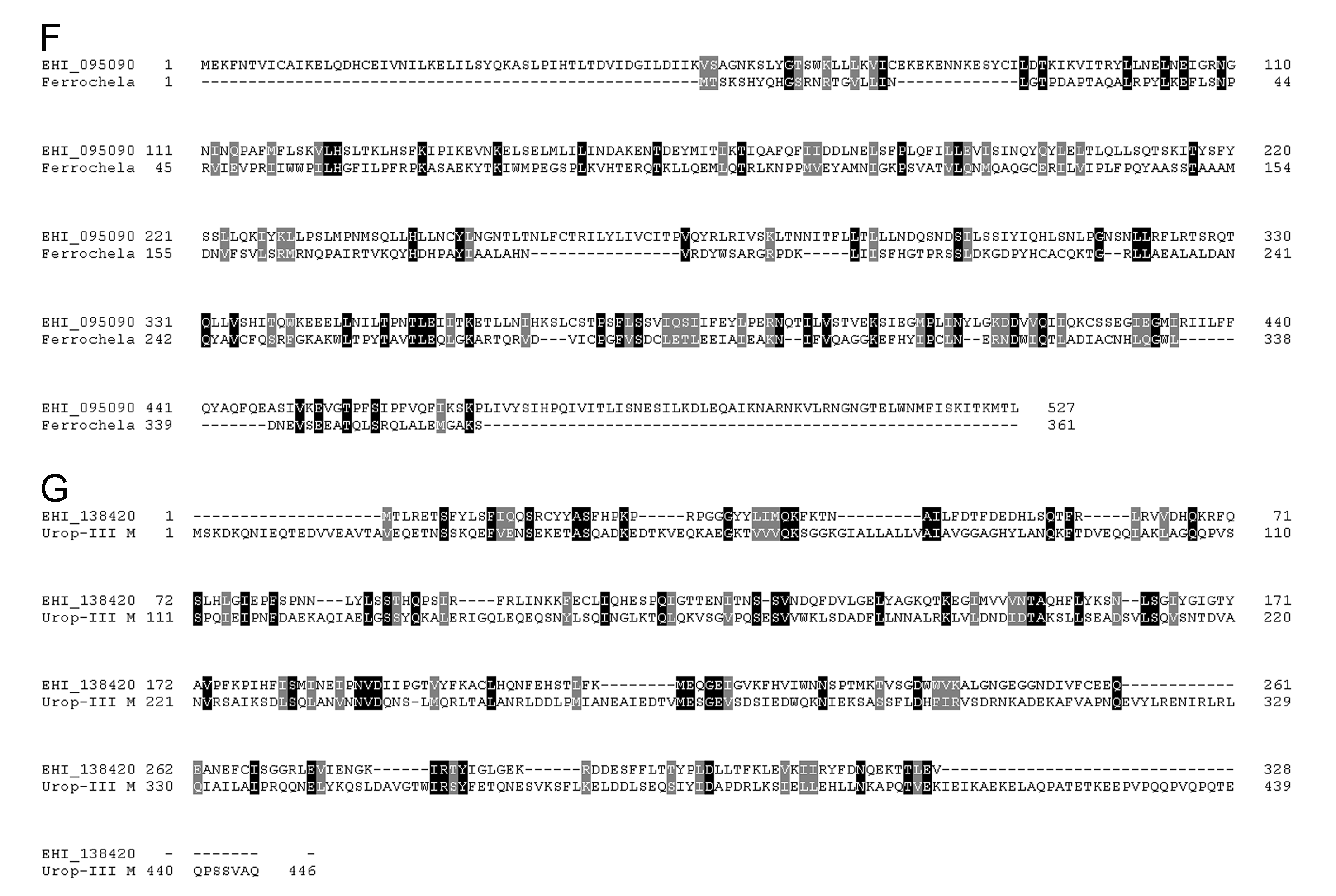


**Figure S3**. **Alignments of selected *E. histolytica* genes.** A) amoebic ComEC orthologs (EHI_169340 and EHI_156240) and ComEC from *Bacillus licheniformis (*WP_003183688.1, 20% similarity), B) amebic P-glycoprotein-5 (EHI_125030) and PvdE from *Pseudomonas aeruginosa (*YP_002440490.1, 33% similarity), C) amoebic MFT (EHI_173950) and MFS1 exporter from *Azotobacter vineldii (*YP_002797373.1, 33% similarity), D) amoebic MFT (EHI_173950) and human FLVCR1 (NP_054772.1), E) amoebic hypothetical protein (EHI_009840) and heme-degrading monooxygenase (isdG) from *Staphylococcus aureus (*NP_645835.1, 38% similarity), F) amoebic hypothetical protein (EHI_095090) and ferrochelatase from *Nitrosomonas sp. (*YP_004294842.1, 27% similarity), and G) amoebic hypothetical protein (EHI_138420) and uroporphyrin-III C-methyltransferase from *Actinobacillus pleuropneumoniae (*WP_005597783.1, 32% similarity). Residues with 100% and 80% homology are highlight in gray and black, respectively. The comparisons were performed using the CLUSTALW alignment tool from the WebExPASy Molecular Biology Server ([http://ca.expasy.org](http://ca.expasy.org/)).

**Table S1. Quantification of iron in TYI-S-33 and TYI-S-33ΔFe** medium.

| **TYI-S-33** |  |  |
| --- | --- | --- |
| Complete | Incomplete (non serum) | Iron in serum |
| 168.9 μM ± 5.5 | 108.5 μM ± 0.1 | 60.4 μM |
| **TYI-S-33ΔFe** |  |  |
| Complete | Incomplete (non serum) | Iron in serum |
| 90.7 μM ± 6.1 | 39.7 μM ± 0.8 | 51 μM |
|  | = | 55.5 μM |
| **Low Iron** |  |  |
| ≈ 122.7 μM | 90.7 μM from TYI-S-33ΔFe | + 32 μM from AFC supplementation |
| **TYI-S-33ΔFe + Hb** |  |  |
|  | 90.7 μM from TYI-S-33ΔFe | + 84 µM from Hb supplementation |

*Footnote:*

The ferrozine method described in the Material and Methods section was used to quantify iron in the TYI-S-33 medium, incomplete TYI-S-33ΔFe medium (no supplementation with AFC, vitamins and serum) and TYI-S-33ΔFe complete (no supplementation with AFC but supplemented with vitamins and serum) medium. Serum accounts for 55.5 µM iron, i.e. the difference between complete and incomplete media. Peptone accounts for 39.7 µM iron, i.e. the level determined in the incomplete TYI-S-33ΔFe medium. AFC accounts for 78 µM iron, i.e. the difference between the complete TYI-S-33 and complete TYI-S-33ΔFe media. AFC: ammonium ferric citrate; Hb: hemoglobin. Values correspond to the mean of experiments performed in triplicate.

**Table S2.** **Differentially expressed genes in iron deficiency condition**.

| Description | AmoebaDB ID | FC | BY | rawp |
| --- | --- | --- | --- | --- |
| **Upregulated** |  |  |  |  |
| Competence protein ComEC | EHI_156240 | 6.5 | 1.01E-10 | 1.33E-15 |
| Acyl-CoA synthetase | EHI_153060 | 5.7 | 6.83E-10 | 1.80E-14 |
| Cysteine proteinase CP-A4 | EHI_050570 | 3.0 | 9.53E-04 | 5.02E-08 |
| AIG1 family protein | EHI_022500 | 2.5 | 2.82E-03 | 2.27E-07 |
| Competence protein ComEC | EHI_169340 | 2.4 | 2.82E-03 | 3.34E-07 |
| Hypothetical protein | EHI_058920 | 2.4 | 7.77E-03 | 1.84E-06 |
| AIG1 family protein | EHI_195260 | 2.3 | 1.41E-02 | 4.23E-06 |
| AIG1 family protein | EHI_115160 | 2.2 | 2.54E-03 | 1.67E-07 |
| EhNO2 | EHI_045340 | 2.2 | 4.67E-02 | 1.96E-05 |
| **Downregulated** |  |  |  |  |
| Monooxygenase protein | EHI_009840 | -3.7 | 3.10E-04 | 1.22E-08 |
| S-adenosylmethionine synthetase | EHI_174250 | -3.5 | 2.82E-03 | 2.96E-07 |
| S-adenosylmethionine synthetase | EHI_004920 | -3.3 | 7.10E-03 | 1.40E-06 |
| Iron sulfur flavoprotein | EHI_022600 | -2.9 | 3.00E-03 | 4.34E-07 |
| S-adenosylmethionine synthetase | EHI_195110 | -2.9 | 1.45E-02 | 4.59E-06 |
| Hypothetical protein | EHI_048140 | -2.8 | 3.42E-03 | 5.40E-07 |
| Iron sulfur flavoprotein | EHI_067720 | -2.6 | 7.77E-03 | 1.83E-06 |
| Hypothetical protein | EHI_022270 | -2.3 | 5.49E-03 | 9.39E-07 |
| Iron sulfur flavoprotein | EHI_103260 | -2.3 | 8.00E-03 | 2.00E-06 |
| Ubiquitin ligase | EHI_104570 | -2.2 | 4.24E-02 | 1.67E-05 |
| Phosphoglycerate dehydrogenase | EHI_060860 | -2.0 | 1.38E-02 | 3.82E-06 |

*Footnote:*

FC: fold-change; BY: The false discovery rate according to Benjamini and Yekutieli multiple testing; rawp: raw the unadjusted *P*-value.

**Table S3.** **Differentially expressed genes in iron deficiency condition with Hb supplementation**.

| Description | AmoebaDB ID | | FC | BY | | | rawp |  |
| --- | --- | --- | --- | --- | --- | --- | --- | --- |
| **Upregulated** |  | |  |  | | |  |  |
| Aldose reductase | EHI_157010 | | 5.6 | 1.12E-12 | | | 2.22E-16 |  |
| Acyl-CoA synthetase | EHI_153060 | | 5.4 | 1.12E-12 | | | 2.22E-16 |  |
| Aldose reductase | EHI_039190 | | 5.1 | 0.00E+00 | | | 0.00E+00 |  |
| Aldose reductase | EHI_107560 | | 5.0 | 0.00E+00 | | | 0.00E+00 |  |
| P-glycoprotein 5 | EHI_125030 | | 4.8 | 0.00E+00 | | | 0.00E+00 |  |
| Hypothetical protein | EHI_151930 | | 4.5 | 0.00E+00 | | | 0.00E+00 |  |
| Hypothetical protein | EHI_023330 | | 4.5 | 0.00E+00 | | | 0.00E+00 |  |
| RIO1 family protein | EHI_170330 | | 4.5 | 4.69E-12 | | | 1.11E-15 |  |
| Cysteine proteinase | EHI_010850 | | 4.5 | 1.12E-12 | | | 2.22E-16 |  |
| EhCP A7 cysteine proteinase | EHI_039610 | | 4.5 | 0.00E+00 | | | 0.00E+00 |  |
| Hypothetical protein | EHI_058920 | | 4.2 | 1.12E-12 | | | 2.22E-16 |  |
| Alcohol dehydrogenase | EHI_088020 | | 4.2 | 1.12E-12 | | | 2.22E-16 |  |
| Competence protein ComEC | EHI_156240 | | 4.1 | 0.00E+00 | | | 0.00E+00 |  |
| Hypothetical protein | EHI_009990 | | 4.1 | 3.51E-10 | | | 1.06E-13 |  |
| Cysteine proteinase 2 precursor | EHI_132650 | | 4.0 | 0.00E+00 | | | 0.00E+00 |  |
| Double strand break repair protein MRE11 | EHI_125910 | | 3.9 | 1.16E-09 | | | 3.99E-13 |  |
| Cysteine synthase CS2 | EHI_160930 | | 3.9 | 1.12E-12 | | | 2.22E-16 |  |
| Cysteine synthase CS1 | EHI_171750 | | 3.8 | 2.79E-09 | | | 1.10E-12 |  |
| Cysteine protease | EHI_144040 | | 3.8 | 2.11E-12 | | | 4.44E-16 |  |
| DEAD/DEAH box helicase | EHI_131080 | | 3.7 | 5.62E-11 | | | 1.55E-14 |  |
| Hypothetical protein | EHI_187790 | | 3.7 | 7.90E-09 | | | 3.53E-12 |  |
| Hypothetical protein | EHI_174580 | | 3.7 | 8.23E-09 | | | 3.79E-12 |  |
| Regulator of nonsense transcripts | EHI_070810 | | 3.7 | 1.09E-10 | | | 3.15E-14 |  |
| Protein kinase | EHI_140330 | | 3.7 | 9.86E-09 | | | 4.67E-12 |  |
| Alcohol dehydrogenase 3 | EHI_160670 | | 3.7 | 1.95E-09 | | | 7.43E-13 |  |
| Hypothetical protein | EHI_075990 | | 3.7 | 1.02E-08 | | | 4.97E-12 |  |
| Hypothetical protein | EHI_169830 | | 3.6 | 1.28E-08 | | | 6.58E-12 |  |
| Ribosomal protein S30 | EHI_023400 | | 3.6 | 1.77E-09 | | | 6.28E-13 |  |
| Glutamyl-tRNA synthetase | EHI_155570 | | 3.4 | 6.92E-10 | | | 2.28E-13 |  |
| Iron sulfur flavoprotein | EHI_138480 | | 3.3 | 5.05E-09 | | | 2.13E-12 |  |
| Eukaryotic translation initiation factor 6 | EHI_006170 | | 3.3 | 3.88E-10 | | | 1.23E-13 |  |
| Hypothetical protein | EHI_159670 | | 3.2 | 1.79E-09 | | | 6.61E-13 |  |
| Cysteine desulfurase (NIF) | EHI_136380 | | 3.2 | 1.02E-06 | | | 6.95E-10 |  |
| P-glycoprotein 5 | EHI_175450 | | 3.2 | 6.00E-09 | | | 2.61E-12 |  |
| Cysteine proteinase | EHI_144050 | | 3.1 | 1.57E-08 | | | 8.49E-12 |  |
| Ribosomal protein S30 | EHI_088600 | | 3.0 | 4.19E-09 | | | 1.71E-12 |  |
| Hypothetical protein | EHI_131560 | | 3.0 | 3.91E-06 | | | 3.24E-09 |  |
| Chaperone clpB | EHI_090840 | | 2.9 | 1.34E-05 | | | 1.64E-08 |  |
| ATP binding cassette | EHI_095820 | | 2.9 | 1.45E-08 | | | 7.66E-12 |  |
| Hypothetical protein | EHI_197520 | | 2.8 | 5.61E-07 | | | 3.69E-10 |  |
| Fe-S cluster assembly protein NifU | EHI_049620 | | 2.7 | 1.26E-06 | | | 9.09E-10 |  |
| Regulator of nonsense transcripts | EHI_110840 | | 2.7 | 8.78E-08 | | | 5.25E-11 |  |
| Hypothetical protein | EHI_148870 | | 2.6 | 3.19E-05 | | | 4.87E-08 |  |
| Hypothetical protein | EHI_114770 | | 2.6 | 6.82E-06 | | | 6.38E-09 |  |
| Hypothetical protein | EHI_112830 | | 2.6 | 2.13E-05 | | | 2.86E-08 |  |
| Transporter major facilitator | EHI_173950 | | 2.5 | 5.35E-08 | | | 3.03E-11 |  |
| Hypothetical protein | EHI_087110 | | 2.5 | 2.45E-04 | | | 7.36E-07 |  |
| Iron containing superoxide dismutase (SOD) | EHI_159160 | | 2.5 | 2.53E-07 | | | 1.63E-10 |  |
| Alcohol dehydrogenase (adh2) | EHI_150490 | | 2.5 | 3.65E-04 | | | 1.33E-06 |  |
| Hypothetical protein | EHI_025880 | | 2.5 | 2.51E-05 | | | 3.47E-08 |  |
| Hydroxylamine reductase | EHI_004600 | | 2.5 | 8.69E-08 | | | 5.03E-11 |  |
| Hypothetical protein | EHI_050590 | | 2.4 | 4.64E-04 | | | 2.01E-06 |  |
| 70 kDa peptidyl prolyl isomerase | EHI_178850 | | 2.4 | 4.71E-04 | | | 2.06E-06 |  |
| Fatty acid elongase | EHI_112870 | | 2.4 | 8.63E-06 | | | 8.97E-09 |  |
| Hypothetical protein | EHI_115400 | | 2.4 | 5.19E-04 | | | 2.45E-06 |  |
| Hypothetical protein | EHI_143090 | | 2.4 | 1.73E-05 | | | 2.23E-08 |  |
| EhCP A5 cysteine proteinase | EHI_168240 | | 2.4 | 1.19E-06 | | | 8.46E-10 |  |
| Malate dehydrogenase | EHI_014410 | | 2.4 | 5.87E-06 | | | 5.33E-09 |  |
| N system amino acid transporter 1 | EHI_050900 | | 2.4 | 8.96E-07 | | | 6.02E-10 |  |
| Malate dehydrogenase | EHI_014410 | | 2.4 | 1.45E-04 | | | 3.42E-07 |  |
| Hypothetical protein | EHI_184560 | | 2.3 | 2.66E-06 | | | 2.10E-09 |  |
| U3 small nucleolar ribonucleo protein protein MPP10 | EHI_048860 | | 2.3 | 7.20E-05 | | | 1.40E-07 |  |
| Aldehyde alcohol dehydrogenase 2 | EHI_024240 | | 2.3 | 9.75E-04 | | | 6.42E-06 |  |
| Hypothetical protein | EHI_095850 | | 2.3 | 8.34E-06 | | | 8.45E-09 |  |
| Serine threonine isoleucine rich protein (STIRP) | EHI_025700 | | 2.3 | 1.54E-05 | | | 1.95E-08 |  |
| Phosphoserine aminotransferase (serC) | EHI_026360 | | 2.2 | 2.11E-07 | | | 1.33E-10 |  |
| Hypothetical protein | EHI_174230 | | 2.2 | 3.15E-04 | | | 1.08E-06 |  |
| RNA binding protein | EHI_151990 | | 2.2 | 1.28E-04 | | | 2.84E-07 |  |
| ATP binding cassette protein | EHI_178580 | | 2.2 | 2.51E-05 | | | 3.47E-08 |  |
| Acyl-CoA synthetase | EHI_131880 | | 2.2 | 1.05E-05 | | | 1.13E-08 |  |
| Iron sulfur flavoprotein | EHI_022600 | | 2.2 | 7.86E-04 | | | 4.73E-06 |  |
| Midasin | EHI_025850 | | 2.2 | 2.90E-03 | | | 3.04E-05 |  |
| Heat shock protein | EHI_156560 | | 2.2 | 3.85E-04 | | | 1.53E-06 |  |
| Kinase, PfkB family | EHI_157120 | | 2.2 | 4.99E-06 | | | 4.34E-09 |  |
| Serine threonine isoleucine rich protein (STIRP) | EHI_004340 | | 2.2 | 3.64E-04 | | | 1.31E-06 |  |
| Eukaryotic translation initiation factor 4 gamma | EHI_044930 | | 2.2 | 6.11E-05 | | | 1.15E-07 |  |
| Hypothetical protein | EHI_062310 | | 2.2 | 5.85E-06 | | | 5.23E-09 |  |
| Nonpathogenic pore forming peptide precursor | EHI_169350 | | 2.2 | 2.28E-04 | | | 6.42E-07 |  |
| Malate dehydrogenase | EHI_165350 | | 2.1 | 2.23E-04 | | | 6.23E-07 |  |
| Heat shock protein70, hsp70A2 | EHI_015390 | | 2.1 | 1.26E-04 | | | 2.78E-07 |  |
| Hypothetical protein | EHI_080880 | | 2.1 | 2.57E-04 | | | 8.02E-07 |  |
| Hypothetical protein | EHI_031640 | | 2.1 | 4.71E-04 | | | 2.08E-06 |  |
| Chaperone clpB | EHI_155060 | | 2.1 | 3.12E-04 | | | 1.07E-06 |  |
| Heat shock protein 101 | EHI_183680 | | 2.1 | 6.79E-04 | | | 3.72E-06 |  |
| Aldehyde alcohol dehydrogenase 2 | EHI_160940 | | 2.1 | 3.17E-03 | | | 3.45E-05 |  |
| Hypothetical protein | EHI_113950 | | 2.1 | 6.86E-04 | | | 3.78E-06 |  |
| Hypothetical protein | EHI_142050 | | 2.1 | 3.32E-05 | | | 5.12E-08 |  |
| Hypothetical protein | EHI_006430 | | 2.1 | 3.23E-04 | | | 1.13E-06 |  |
| P-glycoprotein 5 | EHI_075410 | | 2.1 | 5.60E-04 | | | 2.75E-06 |  |
| Hypothetical protein | EHI_012350 | | 2.1 | 2.60E-04 | | | 8.14E-07 |  |
| ATP binding cassette | EHI_178050 | | 2.0 | 5.40E-05 | | | 9.52E-08 |  |
| Heat shock protein 90 | EHI_196940 | | 2.0 | 7.76E-03 | | | 1.15E-04 |  |
| Hypothetical protein | EHI_022270 | | 2.0 | 3.84E-04 | | | 1.48E-06 |  |
| DNA directed RNA polymerase III subunit | EHI_050830 | | 2.0 | 1.18E-02 | | | 2.04E-04 |  |
| Heat shock protein 101 | EHI_178230 | | 2.0 | 1.45E-03 | | | 1.12E-05 |  |
| Hypothetical protein | EHI_198580 | | 2.0 | 3.46E-05 | | | 5.37E-08 |  |
| Sulfotransferase | EHI_197340 | | 2.0 | 8.12E-05 | | | 1.66E-07 |  |
| Regulator of nonsense transcripts | EHI_035550 | | 2.0 | 4.75E-04 | | | 2.10E-06 |  |
| Hypothetical protein | EHI_002240 | | 2.0 | 7.46E-04 | | | 4.32E-06 |  |
| Hypothetical protein | EHI_034010 | | 2.0 | 2.27E-03 | | | 2.15E-05 |  |
| Zinc finger domain containing protein | EHI_036640 | | 2.0 | 1.16E-03 | | | 7.97E-06 |  |
| Table S3 cont’  Hypothetical protein | EHI_039330 | 2.0 | | | 3.18E-04 | 1.11E-06 | | |
| DEAD box ATP dependent RNA helicase 42 | EHI_197990 | 2.0 | | | 1.86E-03 | 1.54E-05 | | |
| DEAD/DEAH box helicase | EHI_119920 | 2.0 | | | 6.35E-05 | 1.21E-07 | | |
| Proteasome alpha subunit | EHI_090000 | 2.0 | | | 2.28E-03 | 2.20E-05 | | |
| Acetyltransferase | EHI_039180 | 2.0 | | | 2.34E-06 | 1.79E-09 | | |
| Iron sulfur flavoprotein | EHI_067720 | 2.0 | | | 9.92E-04 | 6.59E-06 | | |
| Ribonucleoprotein | EHI_053830 | 2.0 | | | 2.76E-03 | 2.85E-05 | | |
|  |  |  | | |  |  | | |
| Description | AmoebaDB ID | FC | | | BY | rawp | | |
| **Downregulated** |  |  | | |  |  | | |
| Monooxygenase protein | EHI_009840 | 20.6 | | | 1.12E-12 | 2.22E-16 | | |
| Galactose inhibitable lectin | EHI_058330 | 4.1 | | | 6.22E-12 | 1.55E-15 | | |
| Hypothetical protein | EHI_024490 | 4.0 | | | 4.69E-12 | 1.11E-15 | | |
| Alcohol dehydrogenase | EHI_125950 | 3.9 | | | 4.56E-11 | 1.20E-14 | | |
| Fatty acid elongase | EHI_009370 | 2.6 | | | 1.28E-08 | 6.46E-12 | | |
| Methionine gamma lyase | EHI_057550 | 2.6 | | | 1.21E-05 | 1.40E-08 | | |
| Ganglioside gm2 activator protein | EHI_151800 | 2.5 | | | 3.70E-05 | 6.05E-08 | | |
| Aspartate ammonia lyase | EHI_150390 | 2.5 | | | 1.33E-07 | 8.20E-11 | | |
| Hypothetical protein | EHI_028960 | 2.5 | | | 2.59E-05 | 3.72E-08 | | |
| Longevity assurance family protein | EHI_151290 | 2.5 | | | 1.66E-06 | 1.22E-09 | | |
| Hypothetical protein | EHI_111770 | 2.5 | | | 3.59E-08 | 1.99E-11 | | |
| Bacterial transferase hexapeptide family protein | EHI_004840 | 2.4 | | | 2.39E-06 | 1.85E-09 | | |
| Hypothetical protein | EHI_086380 | 2.4 | | | 7.59E-06 | 7.29E-09 | | |
| ADP ribosylation factor | EHI_189960 | 2.4 | | | 4.93E-06 | 4.22E-09 | | |
| Lysozyme | EHI_199110 | 2.4 | | | 8.78E-08 | 5.32E-11 | | |
| Metal dependent hydrolase | EHI_054690 | 2.3 | | | 1.34E-05 | 1.64E-08 | | |
| Hypothetical protein | EHI_022010 | 2.3 | | | 3.69E-05 | 5.88E-08 | | |
| NADP dependent alcohol dehydrogenase | EHI_023110 | 2.3 | | | 3.11E-05 | 4.70E-08 | | |
| Aspartate ammonia lyase | EHI_082270 | 2.3 | | | 1.37E-04 | 3.08E-07 | | |
| Hypothetical protein | EHI_201240 | 2.3 | | | 1.32E-05 | 1.56E-08 | | |
| Hypothetical protein | EHI_005970 | 2.3 | | | 6.30E-06 | 5.80E-09 | | |
| Carbonic anhydrase | EHI_073380 | 2.3 | | | 1.78E-04 | 4.51E-07 | | |
| Lecithin:cholesterol acyltransferase domain containing protein | EHI_064540 | 2.2 | | | 1.09E-05 | 1.20E-08 | | |
| Hypothetical protein | EHI_096790 | 2.2 | | | 2.34E-06 | 1.78E-09 | | |
| Heat shock protein, Hsp20 family | EHI_125830 | 2.1 | | | 3.69E-05 | 5.97E-08 | | |
| Molybdenum cofactor sulfurase putative | EHI_194600 | 2.1 | | | 1.12E-06 | 7.83E-10 | | |
| Methionine gamma lyase | EHI_144610 | 2.1 | | | 3.70E-04 | 1.41E-06 | | |
| DNA directed RNA polymerase II subunit | EHI_056690 | 2.1 | | | 1.86E-05 | 2.42E-08 | | |
| Hypothetical protein | EHI_103520 | 2.1 | | | 3.11E-05 | 4.71E-08 | | |
| Translation initiation factor eIF 5A (eif5A) | EHI_151810 | 2.1 | | | 8.02E-05 | 1.63E-07 | | |
| Lysozyme | EHI_096570 | 2.1 | | | 9.96E-06 | 1.05E-08 | | |
| Lecithin:cholesterol acyltransferase domain containing protein | EHI_020250 | 2.1 | | | 4.80E-05 | 8.28E-08 | | |
| Acetyltransferase, GNAT family | EHI_167080 | 2.1 | | | 8.02E-05 | 1.62E-07 | | |
| Hypothetical protein | EHI_163530 | 2.1 | | | 8.15E-06 | 8.15E-09 | | |
| Phosphoglycerate dehydrogenase | EHI_060860 | 2.1 | | | 1.04E-05 | 1.10E-08 | | |
| Galactose inhibitable lectin small subunit | EHI_159870 | 2.1 | | | 3.85E-04 | 1.51E-06 | | |

Table S3 cont’

| Hypothetical protein | EHI_017590 | 2.0 | 5.92E-04 | 3.05E-06 |
| --- | --- | --- | --- | --- |
| Hypothetical protein | EHI_150130 | 2.0 | 1.27E-05 | 1.49E-08 |
| Amino acid transporter | EHI_072120 | 2.0 | 4.28E-06 | 3.60E-09 |
| Fe hydrogenase | EHI_005060 | 2.0 | 2.54E-05 | 3.54E-08 |
| Hypothetical protein | EHI_133210 | 2.0 | 1.53E-05 | 1.89E-08 |
| Hypothetical protein | EHI_143070 | 2.0 | 4.76E-05 | 8.14E-08 |
| WD domain containing protein | EHI_126260 | 2.0 | 5.57E-05 | 1.01E-07 |
| Transketolase | EHI_157770 | 2.0 | 1.03E-04 | 2.17E-07 |
| Calmodulin | EHI_010020 | 2.0 | 3.20E-06 | 2.57E-09 |
| Hypothetical protein | EHI_187780 | 2.0 | 2.57E-04 | 8.03E-07 |
| Hypothetical protein | EHI_032470 | 2.0 | 2.51E-05 | 3.43E-08 |
| Hypothetical protein | EHI_153090 | 2.0 | 1.09E-05 | 1.22E-08 |
| Protein kinase rad3 | EHI_129730 | 2.0 | 5.57E-05 | 1.00E-07 |
| Hypothetical protein | EHI_109690 | 2.0 | 1.21E-05 | 1.38E-08 |

Table S3 cont’

*Footnote:*

FC: fold-change; BY: The false discovery rate according to Benjamini and Yekutieli multiple testing; rawp: raw the unadjusted *P*-value.

**Table S4. Differentially expressed genes in low iron conditions.**

| Description | AmoebaDB ID | FC | BY | rawp |
| --- | --- | --- | --- | --- |
| **Upregulated** |  |  |  |  |
| Heat shock protein 101 | EHI_156560 | 4.1 | 9.22E-05 | 6.06E-09 |
| Heat shock protein 101 | EHI_183680 | 3.9 | 1.63E-04 | 2.11E-08 |
| Chaperone clpB | EHI_090840 | 3.9 | 1.63E-04 | 2.16E-08 |
| Regulator of nonsense transcripts | EHI_070810 | 3.8 | 1.63E-04 | 2.71E-08 |
| Heat shock protein 101 | EHI_178230 | 3.8 | 2.21E-04 | 4.06E-08 |
| Chaperone clpB | EHI_155060 | 3.5 | 4.22E-04 | 1.48E-07 |
| Heat shock protein 101 | EHI_076480 | 3.3 | 1.16E-03 | 7.60E-07 |
| Glutamyl tRNA synthetase | EHI_155570 | 3.1 | 1.77E-03 | 1.56E-06 |
| Regulator of nonsense transcripts | EHI_110840 | 3.0 | 1.99E-03 | 1.90E-06 |
| S-adenosylmethionine synthetase | EHI_004920 | 2.9 | 4.09E-03 | 8.02E-06 |
| Sulfotransferase | EHI_197340 | 2.8 | 2.32E-04 | 4.88E-08 |
| Alcohol dehydrogenase | EHI_088020 | 2.8 | 8.40E-04 | 5.09E-07 |
| Heat shock protein 101 | EHI_013550 | 2.8 | 7.03E-03 | 1.86E-05 |
| Iron sulfur flavoprotein | EHI_138480 | 2.8 | 7.68E-03 | 2.25E-05 |
| Hypothetical protein | EHI_163240 | 2.7 | 1.16E-02 | 4.37E-05 |
| S-adenosylmethionine synthetase | EHI_174250 | 2.7 | 1.22E-02 | 4.69E-05 |
| Chaperone clpB | EHI_094680 | 2.7 | 1.25E-02 | 4.96E-05 |
| Hypothetical protein | EHI_023330 | 2.6 | 1.16E-02 | 4.36E-05 |
| Heat shock protein 101 | EHI_094470 | 2.5 | 2.06E-02 | 1.11E-04 |
| S-adenosylmethionine synthetase | EHI_195110 | 2.5 | 2.54E-02 | 1.50E-04 |
| Hypothetical protein | EHI_063440 | 2.5 | 2.65E-02 | 1.60E-04 |
| Cysteine synthase CS2 | EHI_160930 | 2.5 | 3.11E-03 | 4.54E-06 |
| Hypothetical protein | EHI_114770 | 2.4 | 1.63E-04 | 2.48E-08 |
| Asparaginyl-tRNA synthetase | EHI_126920 | 2.4 | 2.05E-02 | 1.10E-04 |
| P-glycoprotein 5 | EHI_175450 | 2.4 | 2.83E-03 | 3.91E-06 |
| Hypothetical protein | EHI_184560 | 2.3 | 4.29E-04 | 1.67E-07 |
| Hypothetical protein | EHI_136430 | 2.2 | 1.48E-02 | 6.61E-05 |
| Hypothetical protein | EHI_098440 | 2.2 | 2.37E-03 | 2.47E-06 |
| ATP binding cassette protein | EHI_178580 | 2.2 | 4.83E-02 | 4.09E-04 |
| Regulator of nonsense transcripts | EHI_193520 | 2.2 | 7.61E-03 | 2.18E-05 |
| Hypothetical protein | EHI_008420 | 2.2 | 5.37E-03 | 1.23E-05 |
| Hypothetical protein | EHI_199620 | 2.1 | 1.40E-03 | 1.17E-06 |
| P-glycoprotein 5 | EHI_125030 | 2.1 | 5.39E-03 | 1.24E-05 |
| Hypothetical protein | EHI_159760 | 2.0 | 1.27E-03 | 1.00E-06 |

| Description | AmoebaDB ID | FC | BY | rawp |
| --- | --- | --- | --- | --- |
| **Downregulated** |  |  |  |  |
| Monooxygenase protein | EHI_009840 | 4.4 | 2.18E-05 | 8.60E-10 |
| Actobindin | EHI_158570 | 4.0 | 2.18E-05 | 6.71E-10 |
| Alcohol dehydrogenase | EHI_125950 | 3.6 | 2.45E-07 | 3.22E-12 |
| Hypothetical protein | EHI_048140 | 3.6 | 3.35E-04 | 9.20E-08 |
| Actobindin | EHI_039020 | 3.3 | 1.63E-04 | 2.79E-08 |
| Grainin 2 | EHI_111720 | 3.3 | 1.63E-04 | 1.65E-08 |
| Grainin 2 | EHI_167310 | 3.1 | 1.63E-04 | 2.47E-08 |
| N acetylmuraminidase | EHI_176820 | 3.0 | 4.22E-04 | 1.55E-07 |
| Calmodulin | EHI_010020 | 3.0 | 8.29E-04 | 4.91E-07 |
| Aminoacyl histidine dipeptidase | EHI_042170 | 2.9 | 4.29E-04 | 1.80E-07 |
| Fe-hydrogenase | EHI_005060 | 2.9 | 5.42E-04 | 2.71E-07 |
| Galactose specific adhesin light subunit | EHI_049690 | 2.8 | 3.15E-05 | 1.66E-09 |
| Grainin 1 | EHI_167300 | 2.8 | 3.57E-04 | 1.17E-07 |
| NADP dependent alcohol dehydrogenase | EHI_023110 | 2.7 | 1.27E-03 | 9.25E-07 |
| Nucleoside diphosphate kinase | EHI_104360 | 2.7 | 7.04E-04 | 3.98E-07 |
| Calmodulin | EHI_141840 | 2.4 | 3.57E-03 | 6.17E-06 |
| Hypothetical protein | EHI_092110 | 2.4 | 7.26E-03 | 1.98E-05 |
| Malate dehydrogenase | EHI_014410 | 2.4 | 3.35E-04 | 1.01E-07 |
| Actin binding protein, cofilin/tropomyosin | EHI_186840 | 2.4 | 1.51E-02 | 6.88E-05 |
| Rho GDP exchange inhibitor | EHI_147570 | 2.3 | 2.83E-03 | 3.88E-06 |
| Hypothetical protein | EHI_004550 | 2.3 | 1.27E-03 | 9.41E-07 |
| Aspartate ammonia lyase | EHI_082270 | 2.3 | 1.76E-02 | 8.75E-05 |
| Hypothetical protein | EHI_165280 | 2.2 | 5.42E-04 | 2.69E-07 |
| L myo inositol 1 phosphate synthase | EHI_165270 | 2.2 | 1.27E-03 | 1.00E-06 |
| Hypothetical protein | EHI_053140 | 2.2 | 2.36E-02 | 1.33E-04 |
| LIM zing fnger domain containing protein | EHI_096420 | 2.2 | 3.93E-03 | 7.45E-06 |
| Hypothetical protein | EHI_092680 | 2.2 | 1.68E-03 | 1.46E-06 |
| Hypothetical protein | EHI_169900 | 2.2 | 8.17E-03 | 2.50E-05 |
| Galactose inhibitable lectin | EHI_058330 | 2.2 | 3.35E-04 | 9.34E-08 |
| Hypothetical protein | EHI_058920 | 2.1 | 6.62E-04 | 3.57E-07 |
| Hypothetical protein | EHI_109690 | 2.1 | 3.42E-04 | 1.08E-07 |
| Hypothetical protein | EHI_035100 | 2.1 | 4.22E-04 | 1.54E-07 |
| TolA like protein | EHI_052780 | 2.1 | 2.29E-04 | 4.51E-08 |
| Galactose inhibitable lectin 35 kda subunit | EHI_035690 | 2.1 | 3.50E-03 | 5.89E-06 |
| Hypothetical protein | EHI_055700 | 2.1 | 8.42E-04 | 5.21E-07 |
| Hypothetical protein | EHI_174970 | 2.0 | 2.69E-02 | 1.67E-04 |
| Hypothetical protein | EHI_156220 | 2.0 | 1.99E-03 | 1.91E-06 |
| Endoribonuclease L PSP | EHI_087570 | 2.0 | 1.41E-02 | 6.13E-05 |
| Aspartate ammonia lyase | EHI_150390 | 2.0 | 1.23E-02 | 4.74E-05 |
| ARP2/3 complex 34 kda subunit | EHI_199690 | 2.0 | 2.83E-03 | 3.81E-06 |
| Hypothetical protein | EHI_139380 | 2.0 | 7.26E-03 | 2.00E-05 |
| Hypothetical protein | EHI_161640 | 2.0 | 5.57E-04 | 2.86E-07 |
| Hypothetical protein | EHI_023070 | 2.0 | 2.50E-03 | 3.02E-06 |
| Hypothetical protein | EHI_096320 | 2.0 | 5.69E-04 | 2.99E-07 |
| EF hand calcium binding protein | EHI_023500 | 2.0 | 2.37E-02 | 1.34E-04 |
| Actin related protein 2/3 complex subunit 1A | EHI_045000 | 2.0 | 3.35E-04 | 9.18E-08 |

Table S4 cont’

*Footnote:*

FC: fold-change; BY: The false discovery rate according to Benjamini and Yekutieli multiple testing; rawp: raw the unadjusted *P*-value.

**Table S5. Fold-changes for genes differentially expressed in normal medium + Hb for 2 hours.**

| **Gene description** | **AmoebaDB ID** | **Genebank ID** | **Normal iron + Hb 2h** |
| --- | --- | --- | --- |
| S-adenosylmethionine synthetase | EHI_195110 | XM_001913755 | 1.6 ± 0.7 |
| Glutamyl-tRNA synthetase | EHI_155570 | XM_650693 | 0.9 ± 0.1 |
| Monooxygenase | EHI_009840 | XM_652013 | 1.8 ± 0.5 |

*Footnote:*

The AmoebaDB ID and GenBank ID numbers refer to the gene's accession number in AmoebaDB and NCBI GenBank, respectively; "Normal iron + Hb for 2h" refers to the fold-change in expression in TYI-S-33 medium supplemented with Hb for 2 hours, compared with the normal iron condition (as detected by quantitative real-time PCRs).

**Table S6. Fold-changes for genes differentially expressed in in iron deficiency for 24 hours.**

| **Gene description** | **AmoebaDB ID** | **Genebank ID** | **Normal to iron deficiency 24h** | **Iron deficiency 24h to normal iron 24h** |
| --- | --- | --- | --- | --- |
| S-adenosylmethionine synthetase | EHI_195110 | XM_001913755 | 2.9 ± 0.7 | 1.0 ± 0.2 |
| Glutamyl-tRNA synthetase | EHI_155570 | XM_650693 | 2.5 ± 0.3 | 1.4 ± 0.4 |
| Monooxygenase | EHI_009840 | XM_652013 | -6.3 ± 0.2 | 1.6 ± 0.1 |

*Footnote:*

The AmoebaDB ID and GenBank ID numbers refer to the gene's accession number in AmoebaDB and NCBI GenBank, respectively; “Normal iron to iron deficiency for 24h” refers to the fold-change in expression in TYI-S-33 medium without AFC supplementation for 24 hours, when compared with the normal iron condition (as detected by quantitative real-time PCRs). The trophozoites incubated in iron-deficient medium for 24 hours were recovered and incubated for an additional 24 hours in normal iron medium ("iron deficiency 24h to normal iron 24h"). Gene expression was detected using quantitative real-time PCRs.

**Table S7**. **List of primers used for real time-PCRs.**

| Gene name | **AmoebaDB ID** | Forward | | Reverse | |
| --- | --- | --- | --- | --- | --- |
|  |  | Position | Sequence | Position | Sequence |
| Actobindin | EHI_158570 | 159 | TGAAACTAATGATAGATCAGCTCCAG | 236 | TATTTGGCTTTTGCTTGAATATCAG |
| Actobindin | EHI_039020 | 160 | GAAAGTAATGATAGATCTGCACCAG | 236 | TATTTAGCCTTGGCTTGAATATCAG |
| Grainin 2 | EHI_111720 | 135 | TGAATTAATCAAAGCTCTTCCAATG | 218 | TTTGAAACTTTCTTAGATCCCAATG |
| Grainin 2 | EHI_167310 | 101 | TCCCATTAGTTGAAAGATTAGATGC | 179 | AATTCTCCAATTTCAAGAGTTCCAG |
| Fe-hydrogenase | EHI_005060 | 27 | CTGGTAATCTTGTCGAAGTTATGG | 100 | TTTAATTTTAGCTGATGTAGCTGGTG |
| Monooxygenase | EHI_009840 | 67 | GCAATGCAAAATAAATCAAGAAAAG | 142 | CCCATCTTTGAAGGACTAAATAATG |
| Regulator of nonsense transcript | EHI_110840 | 10 | AAACATTGTGAATATTGTGGAGAAAC | 79 | ACACTCCTGCATTCATAATTTTACC |
| Cell division control protein 42 | EHI_154270 | 330 | TGTTATTGGACTTAAAAATGAAATGG | 420 | GATGTGTCACACTCAAAAACTTCC |
| Uncharacterized protein | EHI_023330 | 1945 | TTCTCAAAAAGACTATGGGACAATC | 2020 | TAGTAACTGGGATTTTGCGTATTTC |
| Glutamyl-tRNA synthetase | EHI_155570 | 122 | ATTGAAGAAGGAATGGTACTTGATG | 197 | TCTCCTTCTTTTAATCCTTTCATAGC |
| S-adenosylmethionine synthetase | EHI_195110 | 732 | AGATGCAGGATTAACAGGAAGAAAG | 811 | TATCAACTTTGCTTGAATCTTTTCC |

*Footnote:*

Position: the relative nucleotide position of the primer's 5’ end, where 0 refers to the first nucleotide of the start codon; Sequence: sequence of the primer from 5’ to 3’. Note that the reverse primer's sequence is reversed and complemented.
